# Bioprocessed Wheat Ingredients: Characterization, Bioaccessibility of Phenolic Compounds, and Bioactivity During *in vitro* Digestion

**DOI:** 10.3389/fpls.2021.790898

**Published:** 2021-12-24

**Authors:** Irene Tomé-Sánchez, Ana Belén Martín-Diana, Elena Peñas, Juana Frias, Daniel Rico, Iván Jiménez-Pulido, Cristina Martínez-Villaluenga

**Affiliations:** ^1^Department of Characterization, Quality and Safety (DCCS), Institute of Food Science, Technology and Nutrition (ICTAN), Spanish National Research Council (CSIC), Madrid, Spain; ^2^Agricultural and Technical Institute of Castile and Leon (ITACyL), Sub-directorate of Research and Technology, Valladolid, Spain

**Keywords:** wheat grain, bran, bioprocessing, oligosaccharides, phenolic compounds, bioactivity, bioaccessibility, digestion

## Abstract

To enlarge the applications of whole wheat grain (WWG) and wheat bran (WB) as functional ingredients in foodstuffs that can promote human health, researchers have explored bioprocessing approaches to improve the bioaccessibility of phenolic compounds from these food matrices and, subsequently, their biological effects. The objective of this study was to compare the composition in nutrients, anti-nutrients, and bioactive compounds of WWG and WB, and their respective bioprocessed products: sprouted wheat (GERM) and WB hydrolysate (stabilized by spray-drying [SPD] and microencapsulated [MEC]). In addition, to evaluate the functional properties of these ingredients, the bioaccessibility of phenolic compounds and their potential antioxidant and anti-inflammatory activities were monitored in different digestion steps. GERM had increased amounts of insoluble dietary fiber, higher diversity of oligosaccharides, and higher concentration of monosaccharides, free phosphorous, and phenolic compounds than WWG. SPD had improved content of soluble dietary fiber, oligosaccharides, monosaccharides, free phosphorous, and phenolic compounds (vs. WB), whereas MEC was mainly composed of protein and had nearly 2-fold lower content of SPD components. All the ingredients showed lower amounts of phytic acid as compared with raw materials. In all samples, hydroxycinnamic acids were the most representative polyphenols followed by minor amounts of hydroxybenzoic acids and flavonoids. Gastrointestinal digestion of GERM, SPD, and MEC revealed high stability of total phenolic compounds in both gastric and intestinal phases. Hydroxycinnamic acids were the most bioaccessible compounds during digestion among the three bioprocessed wheat ingredients studied, although their bioaccessibility varied across ingredients. In this sense, the bioaccessibility of ferulic acid (FA) derivatives increased in GERM with progression of the digestion, while it was reduced in SPD and MEC up to the end of the intestinal phase. Microencapsulation of SPD with pea protein led to generally to lower bioaccessible amounts of phenolic acids. Comparison analysis of biological effects highlighted SPD for its most potent antioxidant effects in the gastrointestinal tract (3 out 4 antioxidant parameters with highest values), while no clear differences were observed with regard to *in vitro* anti-inflammatory activity. Overall, these results support the potential application of GERM, SPD, and MEC as functional and nutraceutical ingredients.

## Introduction

Wheat (*Triticum* spp.), one of the important staple grains in many parts of the world, is a dietary source of starch, fiber, minerals, vitamins, and phytochemicals such as phenolic compounds (phenolic acids, flavonoids, and alkylresorcinols), phytosterols, and sphingolipids, most of them concentrated in outer layers (bran) of the grain (Cheng et al., 2021). For a long time, wheat grain has been applied for white flour production used in the manufacture of a variety of foods, especially baked foods and breakfast cereals. The wheat flour milling industry produces large quantities (about 187 million tons/year) of bran, an inexpensive by-product mostly used for animal feed (Cheng et al., 2021). Based on strong scientific evidence demonstrating that consumption of dietary fiber (DF) is associated with gut health and reduction of cardiovascular diseases and colorectal cancer risk (Cãlinoiu and Vodnar, 2018), whole-wheat grain (WWG) and wheat bran (WB) are being increasingly applied in food production (Cheng et al., 2021). Several mechanisms have been proposed to explain the observed health effects such as bulking capacities of DF, production of short- chained fatty acids due to colonic microbial fermentation of DF, and antioxidant capacity of phenolic compounds (Deroover et al., 2020).

Wheat bioactive compounds with recognized health benefits are naturally trapped in the food matrix and, hence, have low bioavailability (fraction that is absorbed in the gut and reaches the site of action in an active form) that limits their effectiveness to exert physiological functions linked to health effects (bioactivity). Therefore, in the last years, strategies to increase the content of dietary bioactive compounds in WWG and WB while modifying the food structure toward improved delivery of bioactive compounds in the human gastrointestinal tract have attracted the interest of scientists (Mcclements et al., 2015).

Modifications of WWG and WB composition and structure through bioprocessing (germination, fermentation, enzyme treatments) improve not only their nutritional and technological properties but may also positively affect specific health effects (Coda et al., 2015; Lemmens et al., 2019; Onipe et al., 2021). For instance, sprouted grains have positive consumer perceptions due to the existing knowledge of the positive impact of germination on grain nutritional value (Lemmens et al., 2019). Activation of several biochemical events during sprouting of cereal grains results in increased soluble and total phenolic compound content, improves the digestibility of starch, proteins, and lipids, increases soluble DF (SDF), and reduces anti-nutrient content (Lemmens et al., 2019; Tome-Sanchez et al., 2020). Regarding structure, cereal grain germination has resulted in corroded starch granular surface and disrupted crystalline structure, and reduced ordered and helical structures of storage proteins in pulse grains (Xu et al., 2021). All these compositional and structural changes are often related with improved bioaccessibility of bioactive compounds, i.e., with increase in the fraction of bioactive compounds that are released from the food matrix and available for absorption, and enhanced functionality of sprouted grains (Di Stefano et al., 2019; Lemmens et al., 2019). For instance, in our recent study, wheat germination for 7 days at 21°C enhanced the solubilization of phenolic compounds and improved its *in vitro* antioxidant and anti-inflammatory properties (Tome-Sanchez et al., 2020). Preclinical studies support these findings providing evidence on the improvement of potential health benefits of sprouted cereal grains in terms of gut microbiota composition, blood cholesterol and glucose levels, blood pressure, and mineral absorption in rats compared to a control animal group fed with un-germinated grains (Lemmens et al., 2019).

Enzymatic hydrolysis using amylases, xylanases, arabinofuranosidases, cellulases, proteases, and feruloyl esterases has been applied as the main bioprocess to improve WB functional properties. This method has been adopted to enhance the solubilization of arabinoxylan and SDF, release of bound phenolic acids, production of feruloylated oligosaccharides and water-soluble antioxidants, and to increase mineral bioaccessibility (Coda et al., 2015; Bautista-Exposito et al., 2020; Martín-Diana et al., 2021). Our research group has contributed in this field by demonstrating the potential of the combination of thermomechanical (autoclave and high pressure) and enzymatic treatments (Ultraflo XL) to sequentially enhance the free to bound ratio of ferulic acid (FA) and SDF content while improving *in vitro* antioxidant and anti-inflammatory properties of WB (Martín-Diana et al., 2021). Encapsulating phenolic compounds inside food-grade micro-carriers is a safe and an efficient way of increasing their stability, bioactive potential, controlled release, and bioaccessibility in a specific region of the gastrointestinal tract (Huang et al., 2019). With that purpose, we applied atomization and encapsulation techniques after WB enzymatic modification [Martín-Diana, 2021 #7510]. As a result, spray-drying and its further microencapsulation with pea protein (Pisane C_9_) of WB hydrolysate allowed the development of two nutraceutical soluble ingredients rich in SDF and FA with higher antioxidant and anti-inflammatory properties than conventional WB.

Before performing preclinical and clinical trials, it is essential to advance in the knowledge about the health benefits linked to nutraceutical ingredients consumption aimed at elucidating the effect of physiological conditions on the concentration of bioactive compounds and metabolites (Ketnawa et al., 2021), helping to predict their bioaccessibility and bioactivities (antioxidant and anti-inflammatory properties) at different digestion steps. With these considerations, the aim of this study was to evaluate the effect of germination of WWG and hybrid processing of WB (thermal and enzymatic treatments) on nutritional and nutraceutical composition. Finally, changes in the bioaccessibility of phenolic compounds as well as the antioxidant and anti-inflammatory activities of wheat bioprocessed ingredients through different phases of digestion was studied.

## Materials and Methods

### Chemicals, Standards, and Reagents

Fast Blue BB (FBBB) [4-benzoylamino-2,5-dimethoxybenzenediazonium chloride hemi-(zinc chloride) (Rebello et al., 2016), bile extract porcine, pancreatin from porcine pancreas, pepsin from porcine gastric mucosa, α-amylase from human saliva, 2,2′-azinobis 3-ethylbenzothiazoline-6-sulfonic acid (ABTS), 2,2′-diazobis-(2-aminodinopropane)-dihydrochloride (AAPH), fluorescein, 2,2-diphenyl-1-picrylhydrazyl (DPPH) were purchased from Sigma-Aldrich, Co. (St. Louis, MO, United States). Standards of chlorogenic acid, 2-coumaric acid, 2,4-dihydroxybenzoic acid, 4-hydroxybenzoic acid, vanillic acid, and vanillin were provided by Extrasynthese (Lyon, Genay Cedex, France). Standards of 6-hydroxy-2,5,7, 8-tetramethyl-2-carboxylic acid (Trolox), gallic acid, caffeic acid, ferulic acid, vitexin, D-(+)-glucose, D-(+)-galactose, D-(+)-xylose, D-(–)-arabinose, and stachyose were acquired from Sigma-Aldrich, Co. (St. Louis, MO, United States). Standards of 3^3^-α-L-plus 2^3^-α-L-arabinofuranosyl-xylotetraose (XA^3^XX/XA^2^XX), 2^3^,3^3^-di-α-L-arabinofuranosyl-xylotriose (A^2, 3^XX), and xylotriose were obtained from Megazyme (Wicklow, Ireland). Raffinose and cellobiose standards were supplied by Merck (Darmstadt, Germany).

### Materials

Whole wheat grain (WWG) (*Triticum aestivum* L., var. Berdún) and WB (*Triticum aestivum* L. var. Craklin) of < 800 μm particle size were harvested in La Mudarra (2018–2019 crop year) and procured from Emilio Esteban, S.A. (Emesa S.A., Valladolid, Spain). They were stored in vacuum-sealed plastic bags at 20 ± 2°C in darkness until they were used. Pisane C_9_ pea protein was obtained from Innovafood (Barcelona, Spain) and showed the following composition according to the product specification sheet: 0.7/100 g of carbohydrates, 1.4/100 g of dietary fiber, 9/100 g of fat of which saturated 2.1/100 g, 81.7/100 g of protein, and 5/100 g of salt. Commercial food-grade enzyme Ultraflo XL was supplied by Novozymes (Bagsværd, Copenhagen, Denmark). Enzymatic activities of Ultraflo XL include those of cellulase, endo-1,4-β-xylanase, α-L-arabinofuranosidase, and feruloyl esterase (Bautista-Exposito et al., 2020). Murine RAW 264.7 macrophages were supplied by the American Type Culture Collection (ATCC^®^ TIB-71TM; Rockville, MD, United States). Dulbecco's modified Eagle's medium (DMEM) was acquired from Lonza Group (Lonza, Madrid, Spain). Penicillin, streptomycin, and fetal bovine serum (FBS) were purchased from Hyaclone (GE Healthcare, Logan, UT, United States). Lipopolysaccharide (LPS) from *Escherichia coli* O55:B5 was obtained from Sigma-Aldrich, Co. (St. Louis, MO, United States). The Cell Titer 96^®^ AQ_ueous_ One Solution Proliferation Assay kit and murine interleukin (IL)-6 and tumor necrosis factor-alpha (TNF-α) enzyme-linked immunosorbent assay (ELISA) kits were purchased from Promega (Biotech Ibérica, Madrid, Spain) and Diaclone (Besacon Cedex, France), respectively.

### Production of Sprouted Wheat Grains

Germination of WWG was achieved as reported earlier (Tome-Sanchez et al., 2020), with slight modifications. Shortly, WWG (50 g) samples were soaked with 300 ml of 0.1% sodium hypochlorite (ratio 1:6, w:v) for 30 min. Subsequently, they were washed and soaked for 4 h with sterile tap water (ratio 1:6, w:v). The soaked WWG samples were spread uniformly in plastic trays over a steel grid with a moist filter paper and coated with a second moist filter paper, which were previously sterilized. Afterward, seeds were allowed to germinate in an incubator with a water circulation system (Snijders Scientific, Tilburg, The Netherlands). Frequently, the seeds were damped with sterile tap water in order to maintain relative humidity (> 90%). Sprouting was performed without light at 21°C for 7 days in duplicate. These germination parameters were determined based on our earlier optimization study that aimed to maximize soluble phenolic content in addition to anti-inflammatory and antioxidant activity in sprouted wheat flour (GERM) (Tome-Sanchez et al., 2020). The WWG samples were milled (Taurus, Oliana, Spain) and included as control. GERM was freeze-dried (Virtis Company, INC Gardiner, NY, United States), ground (Taurus, Oliana, Spain), and meshed using a 0.3-mm screen. Both powders were stored under vacuum at 4–8°C for additional analysis.

### Production of Spray-Dried and Microencapsulated Wheat Bran (WB) Hydrolysate

The spray-dried wheat bran hydrolysate (SPD) was produced following a multistep processing route as previously described (Martín-Diana et al., 2021). A quantity of 100 g of WB soaked in 2 L deionized water (1:20, w:v) was autoclaved at 115°C, 1.2 × 10^5^ Pa for 15 min (Ilpra Plus 100; Ilpra Systems, Barcelona, Spain). Optimal processing conditions during enzymatic hydrolysis of WB were identified in our previous study (Bautista-Exposito et al., 2020). Malic acid was used to reach a pH of 5. Quickly, Ultraflo XL was incorporated at 1% (ratio enzyme:WB, w:w) and then placed in a temperature-controlled water bath (47°C, 20 h) with a magnetic stirrer (Unitronic Vaivén C; Selecta, S.A., Spain) at 1,000 rpm. Enzyme activity was stopped by heat inactivation using a water bath at 95°C for 10 min. WB soluble fraction was collected after removing insoluble particles by filtration using nylon membranes of 0.2 mm pore size.

Spray-drying of the WB hydrolysate (SPD) was carried out in a MM basic rotary spray-dryer (GEA Mobile Minor™, Düsseldorf, Germany) composed of a chamber coupled to a peristaltic pump (Watson Marlow 520S, Wilmington, MA, United States). Spray-drying was performed at 0.6 MPa (compressed air pressure) with a flow rate of 0.78 L/h and an air inlet/outlet temperature of 130/85°C, according to Martín-Diana et al. (2021). Microencapsulated WB hydrolysate (MEC) was obtained under the abovementioned conditions after a mixing step of SPD with pea protein (ratio 1:1, SPD:Pisane C_9_). Afterward, the spray-dried powders were collected in a cyclone placed outside the dryer and stored at 4–8°C until further use.

### Simulated Gastrointestinal Digestion

The INFOGEST 2.0 method (Brodkorb et al., 2019) was performed to simulate the gastric and intestinal phase digestion of GERM, SPD, MEC, and Pisane C_9_. Before the *in vitro* digestion, enzyme activities, and bile concentration were determined. Briefly, 3 g of the sample were mixed (ratio 1:1, w:v) with a simulated salivary fluid containing salivary amylase (75 U/ml) and calcium chloride (0.3 M) for 2 min at 37°C in a Büchi B-491 (Marshall Scientific, Hampton, NH, United States) heating bath. Subsequently, pH value was quickly adjusted to 3 by adding HCl (1 M). Oral bolus was diluted (ratio 1:1, v:v) with simulated gastric containing a pepsin solution (2,000 U/ml) and calcium chloride (0.3 M). In order to finish the gastric phase, pH was adjusted to 7 with sodium hydroxide (1 M) and then a simulated intestinal fluid containing calcium chloride (0.3 M), pancreatin (800 U/ml) and bile (10 mmol/L) were added to gastric chyme (ratio 1:1, v:v). Both gastric and intestinal phases were incubated for 2 h at 37°C and 150 rpm in a G25-controlled environment incubator shaker (New Brunswick Scientific Co, Inc. Edison, NJ, United States). Finally, enzymes were inactivated by heating in a water bath (95°C for 10 min). Digestion phases were freeze-dried (Virtis Company, INC Gardiner, NY, United States) and stored at 4–8°C until additional analysis. All the phases were performed in duplicate.

### Chemical Composition of Wheat Nutraceutical Ingredients

Determination of total starch, dietary fiber, β-glucan, and phytic acid/free phosphorus content was carried out using K-TSTA-100A, K-RINTDF, K-BGLU, and K-PHYT assay kits (Megazyme, Wicklow, Ireland), respectively. Total nitrogen, determined using the Dumas method with a nitrogen analyzer (LECO Corp., St. Joseph, MI, United States), was converted to total protein by conversion factor 6.25. All analyses were performed in duplicate and expressed as g per 100 g of dry weight (d.w.).

#### Monosaccharide Analysis

A quantity of 50–100 mg of the sample powder was mixed with 1 ml of deionized water and homogenized. After incubation for 1 h at 40°C and 2,000 rpm in a thermomixer (ThermoMixer Compact; Eppendorf, AG, Hamburg, Germany), the mixture was centrifuged for 10 min at 21°C and 13,000 rpm (Centrifuge 5424 R; Eppendorf AG, Hamburg, Germany). Deionized water was added to the collected supernatant until a final volume of 1 ml was reached. Monosaccharide extracts were stored at −20°C until they were used.

Monosaccharide composition was determined by high-performance anion-exchange chromatography (HPAEC) in water-soluble extracts of WWG flour, WB, and derived wheat ingredients (GERM, SPD, and MEC). The samples were injected into an ion chromatographic system, which comprises an 800 Dosino dispenser with a Bioscan module coupled to a degasser, and a pulse amperometric detector (Metrohm, Heriau, Switzerland). Monosaccharide separation was performed in a Hamilton RCX-30 column (4.1 mm × 250 mm, 7 μm) with sodium hydroxide (120 mM) as mobile phase at a flow rate of 1 ml/min. Column temperature and injection volume were maintained at 30°C and 20 μl, respectively. The Metrodata IC Net 2.3. software was used for data acquisition and processing. Monosaccharide identification was carried out by comparison of retention time (RT) and sample spike with authentic standards. Calibration curves of a multi-standard with good linearity were used for the quantification of monosaccharides (*R*^2^ > 0.99). Results were expressed as mean ± standard deviation of two independent replicates (g per 100 g d.w.).

#### Extraction and Quantification of Oligosaccharides

A sample of 50 mg was accurately weighed in a 1.5-ml polypropylene microtube, and then 1 ml of 50% ethanol was added and vigorously mixed by vortexing. Then, the sample was incubated at 70°C for 30 min and 2,000 rpm in ThermoMixer Compact Shaker (Eppendorf, AG, Hamburg, Germany). The mixture was centrifuged for 7 min at 4°C and 10,000 g (Centrifuge 5424 R; Eppendorf AG, Hamburg, Germany). The supernatant was carefully collected, and extraction was performed once again. Afterward, both supernatants were mixed and adjusted to a final volume of 5 mL with 50% ethanol (v:v). The mixture was filtered through a 0.45-μm nylon syringe filter and stored at −20°C until further analysis.

Determination of oligosaccharides was performed with HPLC-ESI-QTOF-MS operating in negative mode with the same equipment described in section Determination of Phenolic Profile and Bioaccessibility by HPLC-ESI-QTOF-MS. Soluble oligosaccharide extracts were injected into a Hypercarb Thermo-Teknokroma (100 mm × 2.1 μm × 5 μm) (Teknokroma, Barcelona, Spain) column coupled to a guard column at 30°C. Injection volume was set at 10 μl, and the flow rate was 0.4 mL/min. The mobile phase consisted of ammonium formate (5 mM) (solvent A) andacetonitrile (solvent B), using a gradient elution from 5 to 10% B (5 min); from 10 to 50% B (15 min); from 50 to 5% B (10 min). A time of 10 min was used before the following injection. Mass range was 100–1,000 (*m/z*). The electrospray ionization source conditions were: drying gas (N_2_) flow rate, temperature, and nebulizer pressure, 12 L/min, 350°C, and 45 psi, respectively, and sheath gas temperature and flow rate, 300°C and 6.5 L/min, respectively. Capillary voltage was set at 4 kV, and nozzle and fragmentation, at 0 and 200 V, respectively. Collision energy was fixed at 20 V for targeted MS/MS experiments. MassHunter Data Acquisition (version B.05.00) and Qualitative Analysis (version B.07.00) Workstation (Agilent Technologies, Waldbroon, Germany) were used for data acquisition and processing. Calibration curves of authentic standards were used (0–250 μg/ml, *R*^2^ > 0.96). Data were expressed as mean ± standard deviation of three independent replicates (mg per 100 g d.w.). The relative amount of each degree of polymerization of soluble oligosaccharides tentatively identified (%) was calculated as a percentage of the sum of the areas of extracted precursor ions (*m/z*) with the same degree of polymerization relative to the total area of extracted oligosaccharides.

#### Extraction and Quantification of Total Soluble Phenolic Compounds (TSPCs)

In order to avoid interferences, TSPCs were analyzed by FBBB reaction according to Pico et al. (2020), with slight modifications. A quantity (50–100 mg) of the milled sample was extracted with 1 ml of 80% methanol in 0.1% formic acid. The sample was vortexed and then incubated for 15 min at 30°C and 2,000 rpm (ThermoMixer Compact; Eppendorf AG, Hamburg, Germany). Subsequently, the sample was centrifuged for 5 min at 5°C and 10,000 rpm (Centrifuge 5424 R; Eppendorf AG, Hamburg, Germany). The sample solution was collected, and a second extraction cycle was performed with 1 ml of 70% acetone in 0.1% formic acid. Both methanolic and acetone extracts were combined and increased to 2 ml with deionized water. A volume of 1 mL of TPSC extract was mixed with 100 μL of a freshly prepared FBBB reagent (0.1% in distilled water) and vortexed for 1 min. Immediately, the extract solution was shaken after adding 100 μL of 5% NaOH and allowed to incubate for 120 min in darkness at room temperature (20 ± 2°C). Finally, 200 μl of the incubated mixture was placed in a 96-well-plate, and absorbance was measured in triplicate at 420 nm using a Synergy HT (BioTek Instruments, Winooski, VT, United States) microplate reader. Quantification of TSPCs was performed with linear calibration curves of gallic acid (0–225 μg/ml) and showed good linearity (*R*^2^ > 0.99). All analyses were performed in duplicate. Data were expressed as g of gallic acid equivalents (GAEs) per 100 g of sample d.w.

### Determination of Phenolic Profile and Bioaccessibility by HPLC-ESI-QTOF-MS

Determination of individual phenolic compounds was performed using an Agilent (Agilent Technologies, Santa Clara, CA, United States) system, which includes an Agilent 1200 series liquid chromatograph (LC) coupled with a G1315B diode array detector (DAD) and an Agilent G6530A accurate-mass quadrupole-time of flight mass spectrometer with an electrospray-ionization source with JetStream technology (HPLC-ESI-QTOF-MS). Before being analyzed, phenolic extracts, as prepared in section Extraction and Quantification of Total Soluble Phenolic Compounds (TSPC), were centrifuged at 4°C and 10,000 rpm. Chromatographic separation of the phenolic extracts (20 μl) was performed at 40°C using an Agilent ZORBAX Eclipse XDB-C18 (4.6 mm × 150 mm × 5 μm) (Agilent Technologies, Santa Clara, CA, United States) analytical column, and DAD chromatograms were acquired at 280, 320, 360, and 520 nm. The mobile phases and gradient program, as well as MS and MS/MS runs, were set as previously described (Martín-Diana et al., 2021). Data acquisition and processing software used were MassHunter Data Acquisition (version B.05.00) and Qualitative Analysis (version B.07.00) Workstation (Agilent Technologies, Waldbroon, Germany). Calibration curves of standards were used for quantification (0–25 μg/ml, *R*^2^ > 0.98). Data were expressed as mean ± standard deviation of two independent replicates (mg per 100 g sample d.w.).

Bioaccessibility (%) is defined as the ratio between the concentration of phenolic compounds released in the simulated digestion compared to the concentration of phenolic compounds in the non-digested functional ingredient.


$$\begin{array}{l} \text{Bioaccessibility}\text{ (\%) }=\\ (\frac{\begin{array}{l} \text{Concentration of compond}\\ \text{ released during digestion} \end{array}}{\begin{array}{l} \text{Concentration of compond in the}\\ \text{ food matrix before digestion} \end{array}})\text{ × }100 \end{array}$$

### Total Antioxidant Capacity (TAC)

#### DPPH Radical Scavenging Activity

2,2-Diphenyl-1-picrylhydrazyl (DPPH) radical scavenging potential was determined according to the modification of the method previously described by Brand-Williams et al. (1995). Briefly, a DPPH solution was dissolved with absolute methanol (120 μM). A final volume of 250 μl containing wheat extract, milliQ water, and DPPH solution (ratio 1:4:5, v:v:v) was added on a 96-well-microplate. The absorbance was recorded at 525 nm for 30 min using a microplate reader (FLUOstar Omega; BMG, Ortenberg, Germany). The calibration curve of Trolox solution (7.5–240 μM) was determined. Results were expressed as μmol Trolox equivalents (TE) per 100 g d.w.

#### Oxygen Radical Absorbance Capacity (ORAC)

The ORAC of samples was determined based on a modified procedure described by Ou et al. (2001). The samples, standards, and blanks were diluted in a phosphate buffer (10 mM, pH 7.4). The intensity of the fluorescence was recorded for 2.5 h using a FLUOstar Omega (BMG, Ortenberg, Germany) microplate reader. The wavelengths of excitation and emission were 485 nm and 520 nm, respectively. A Trolox standard curve (7.5–240 mM) was prepared. Results were calculated using the net area under the curves, between the blank and the sample. The ORAC value was expressed as μmol TE per 100 g d.w.

#### ABTS Radical Cation Scavenging Activity

The antioxidant activity of the extracts was also determined by the ABTS^+^procedure (Miller and Rice-Evans, 1997). Briefly, a 7-mM ABTS aqueous solution was mixed with 2.45 mM potassium persulfate at 2:1 ratio (v:v) and kept in the dark at room temperature overnight before use. The ABTS^+^solution was diluted with a 100-mM phosphate buffer solution (pH 7.4) until absorbance at 734 nm reached a value of 0.7 ± 0.02 and equilibrated at 30°C. Ten picoliters of the samples or Trolox standards (at 20–800 μM concentrations) were dissolved in 190 μl of the ABTS^+^solution, and absorbance (at 734 nm) kinetic was measured after initial shaking every 1 min up to 10 min at 30°C using a microplate reader (FLUOstar Omega; BMG, Ortenberg, Germany). Antioxidant activity was expressed as μmol TE per 100 g d.w.

#### Ferric-Reducing Antioxidant Power (FRAP)

The FRAP activity of the samples was determined using a protocol according to [(Benzie and Strain, 1996) #27]. The FRAP reagent was prepared by mixing 300 mM acetate buffer (pH 3.6), 10 mM TPTZ (2,4,6-tripyridyl-*s*-triazine) in 40 mM HCl, and 20 mM FeCl_3_·6H_2_O in a relative proportion of 10:1:1 (v:v:v). The FRAP reagent was prepared fresh daily and was warmed to 37°C prior to use. A volume of 20 μl of H_2_O (blank), FeSO_4_ (400 μM – 3 mM) standard solution or sample extracts was mixed with 1.9 ml of the FRAP reagent. The mixture was incubated at 37°C for 2 h, and the absorbance was read at 593 nm using a FLUOstar Omega (BMG, Ortenberg, Germany) microplate reader. The solution without extract was used as a blank. Data were expressed as mmol Fe^+2^ equivalents per 100 g d.w.

### Determination of Anti-inflammatory Activity

Cell viability and anti-inflammatory activity of undigested and digested wheat ingredients and Pisane C_9_ were determined in murine RAW 264.7 macrophages as previously reported (Martín-Diana et al., 2021), with minor modifications. The inflammatory response of treated cells was induced by the addition of 40 ng/ml of LPS. Positive control was LPS-treated cells without sample extracts. In both assays, the cells were treated with increasing concentration of wheat and Pisane C_9_ extracts (0.05, 0.1, and 0.5 mg/ml and 0.025, 0.05, and 0.25 mg/ml of DMEM supplemented with 100 U/ml of penicillin, 100 mg/L of streptomycin, and 0.1% of FBS, respectively). Absorbance was determined on a Synergy HT (Biotek Instruments, VT, United States) microplate reader at 490 and 450 nm. One hundred percent viability of cell was determined with regard to the untreated cells. Anti-inflammatory activity was calculated as percentage inhibition relative to positive control. Data represent the mean ± standard deviation of five biological replicates. All analyses were carried out in duplicate.

### Statistical Analysis

The data were expressed as mean ± standard deviation. Statgraphics Centurion XVIII (Statgraphics Technologies, The Plains, VA, United States) was used to perform all the statistical analyses. Analysis of variance (ANOVA) and *post-hoc* Duncan's test were carried out to determine statistical differences between mean values.

## Results

### Chemical Characterization

The chemical composition of WWG, WB, and derived products is shown in Table 1. Although starch was the predominant component in both WWG and GERM, a significant reduction (*p* ≤ 0.05) was observed after germination (from 60.96 ± 3.9 to 26.11 ± 1.39 g/100 g). However, germination led to a significant increase in total dietary fiber (TDF) and insoluble dietary fiber (IDF) contents (1.24- and 1.54-fold increase, respectively), with no significant differences in soluble dietary fiber (SDF) and a significant reduction in β-glucan content (22.08%). Although GERM showed similar SDF content compared to WWG, higher diversity of oligosaccharides was detected in the former (Supplementary Material 1). Compared to WWG, raffinose and sucrose were reduced up to undetectable values in GERM, whereas cellobiose was detected in quantifiable amounts (2.28 ± 0.21 g/100 g). A significant increase was observed for total mono- and disaccharides after germination (7.94-fold increase), having an order of abundance of glucose > cellobiose > fructose > arabinose > galactose. The Protein content of WWG (12 g/100 g) remained unaltered after sprouting. While phytic acid content decreased (32.89% reduction), free phosphorous values were higher (8-fold increase) in GERM than in WWG. Regarding phenolic compounds, a significantly higher amount of TSPC and soluble FA isomers was observed in GERM (2.69- and 1.25-fold increase, respectively) compared to WWG.

**Table 1:** Nutritional and nutraceutical composition of whole wheat grain (WWG)- and wheat bran (WB)-derived ingredients.

**Components (g 100/g d.w.)**	**WWG**	**GERM**	**WB**	**SPD**	**MEC**
*Starch*	60.96 ± 3.90^b^	26.11 ± 1.39^a^	19.13 ± 0.56^c^	10.28 ± 0.72^b^	1.39 ± 0.04^a^
Total insoluble dietary fiber	10.62 ± 1.48^a^	16.35 ± 0.78^b^	37.83 ± 0.40^a^	nd	nd
Total soluble polysaccharides	3.74 ± 0.53^a^	4.52 ± 0.55^a^	5.90 ± 0.40^a^	9.74 ± 1.34^b^	12.03 ± 0.97^b^
β-glucan	0.77 ± 0.04^b^	0.60 ± 0.09^a^	1.95 ± 0.10^b^	2.50 ± 0.23^c^	1.35 ± 0.15^a^
Total soluble oligosaccharides	4.85 ± 1.08^a^	3.05 ± 0.17^a^	11.63 ± 0.03^a^	21.91 ± 3.05^b^	9.13 ± 2.32^a^
Raffinose	0.88 ± 0.00	nd	1.42 ± 0.17^a^	1.38 ± 0.23^a^	0.92 ± 0.14^a^
Stachyose	< LOQ	< LOQ	0.14 ± 0.03^c^	0.11 ± 0.02^b^	0.04 ± 0.01^a^
Xylotriose	nd	nd	nd	4.78 ± 0.59^b^	2.98 ± 0.29^a^
XA^3^XX/XA^2^XX	nd	nd	nd	0.09 ± 0.02^b^	0.01 ± 0.01^a^
A^2, 3^XX	nd	nd	nd	0.97 ± 0.12^b^	0.65 ± 0.03^a^
FAXX	nd	nd	nd	0.10 ± 0.08^a^	0.07 ± 0.00^a^
*Total dietary fiber*	19.22 ± 0.14^a^	23.92 ± 1.50^b^	55.36 ± 0.03^c^	31.65 ± 1.70^b^	21.17 ± 1.35^a^
Glucose	0.62 ± 0.02^a^	8.03 ± 0.27^b^	1.19 ± 0.01^a^	4.40 ± 0.21^c^	2.35 ± 0.06^b^
Galactose	0.05 ± 0.01^a^	0.10 ± 0.01^b^	0.11 ± 0.02^a^	0.23 ± 0.02^b^	0.11 ± 0.05^a^
Arabinose	0.03 ± 0.00^a^	0.21 ± 0.01^b^	0.04 ± 0.00^a^	1.09 ± 0.05^c^	0.51 ± 0.01^b^
Xylose	< LOQ^*^	< LOQ^*^	< LOQ^*^	0.70 ± 0.05^b^	0.33 ± 0.04^a^
Fructose	0.43 ± 0.03^a^	2.05 ± 0.04^b^	1.05 ± 0.07^a^	2.18 ± 0.09^c^	1.18 ± 0.06^b^
Sucrose	0.18 ± 0.00	< LOQ^*^	0.17 ± 0.01^a^	1.12 ± 0.06^c^	0.75 ± 0.04^b^
Maltose	nd	nd	nd	9.34 ± 0.31^b^	6.49 ± 0.78^a^
Cellobiose	nd	2.28 ± 0.21	nd	1.03 ± 0.09^b^	0.51 ± 0.06^a^
*Total di- and monosaccharides*	1.31 ± 0.04^a^	10.40 ± 0.27^b^	2.56 ± 0.05^a^	20.10 ± 0.39^c^	12.23 ± 0.80^b^
*Protein*	12.17 ± 1.21^a^	12.07 ± 1.14^a^	15.62 ± 0.09^b^	10.36 ± 0.18^a^	39.18 ± 0.87^c^
*Phytic acid*	0.76 ± 0.02^b^	0.51 ± 0.01^a^	2.82 ± 0.07^c^	0.85 ± 0.10^a^	1.49 ± 0.08^b^
*Free phosphorous*	0.01 ± 0.00^a^	0.08 ± 0.00^b^	0.06 ± 0.00^a^	1.15 ± 0.06^c^	0.72 ± 0.03^b^
**Phenolic compounds**					
TSPC (g GAE/100 g d.w.)	0.16 ± 0.01^a^	0.43 ± 0.01^b^	0.49 ± 0.01^a^	0.93 ± 0.02^c^	0.60 ± 0.01^b^
Soluble FA isomers (mg/100 g d.w.)	1.25 ± 0.04^a^	1.56 ± 0.20^b^	4.03 ± 0.29^a^	177.81 ± 1.21^c^	104.98 ± 1.39^b^

*Data are mean values ± standard deviation of at least three replicates. Different letters in the same row indicate significant differences among the mean values of different treatments (WWG vs. GERM; WB vs. SPD vs. MEC) (one-way analysis of variance, ANOVA, post-hoc Duncan's test, p ≤ 0.05). A^2, 3^XX, 2^3^,3^3^-di-α-L-arabinofuranosyl-xylotriose; XA^3^XX/XA^2^XX, 3^3^-α-L-arabinofuranosyl-xylotetraose/3^3^-α-L-plus 2^3^-α-L-arabinofuranosyl-xylotetraose; d.w., dry weight; FA, ferulic acid; FAXX, O-(5-O-feruloyl-α-L-arabinofuranosyl)-(1-3)-O-β-D-xylopyranosyl-(1-4)-D-xylopyranose; GAE, gallic acid equivalents; GERM, germinated whole-wheat grain; SPD, hydrolyzed WB spray-dried at 130°C; MEC, hydrolyzed WB spray-dried at 130°C and microencapsulated with Pisane C_9_; LOQ, limit of quantification; nd, not detected; TSPC, total soluble phenolic compounds; WB, wheat bran; WWG, whole wheat grain*.

* *LOQ = 0.1 μg/mL*.

A significant reduction in starch values was observed in SPD and MEC when compared to WB (1.86- and 13.76-fold decrease, respectively; Table 1). This effect was also observed in TDF where the values decreased at least twice in SPD and MEC compared to WB (1.75- and 2.61-fold decrease, respectively). Unlike WB where IDF was the most abundant compound, SPD mainly constituted of SDF (31.65 ± 1.70 g/100 g). Total soluble polysaccharides in SPD and MEC were 1.65- and 2.04-fold higher, respectively, than in WB, whereas amounts of total soluble oligosaccharides and β-glucan were 88.39 and 28.21% higher in SPD than in WB, respectively. Nevertheless, no significant differences were observed in total soluble oligosaccharides, and β-glucan content was significantly reduced from 1.95 ± 0.10 g/100 g in WB to 1.35 g/100 g in MEC. With regard to oligosaccharide content, stachyose was significantly reduced in both powders, and no significant differences were observed in raffinose when compared to WB. Conversely, the applied processing technologies favored the release of xylotriose, 3^3^-α-L-arabinofuranosyl-xylotetraose/3^3^-α-L-plus-2^3^-α-L-arabinofuranosyl-xylotetraose, 2^3^,3^3^-di-α-L-arabinofuranosyl-xylotriose, and *O*-(5-*O*-feruloyl-α-L-arabinofuranosyl)-(1-3)-*O*-β-D-xylopyranosyl-(1-4)-D-xylopyranose, and other unidentified oligosaccharides (Supplementary Material 1). Regarding monosaccharide content, a higher amount was found in SPD and MEC than in WB (7.85- and 4.78- fold increase, respectively). Unlike WB, both SPD and MEC contained significant amounts of maltose and cellobiose, where maltose was the major sugar, followed by smaller amounts of glucose, fructose, sucrose, arabinose, cellobiose, xylose, and galactose (decreasing order). Because Pisane C_9_ (81.7% of protein) was used as encapsulating agent, the main compound of MEC was protein showing a 23.56% higher concentration than WB. In contrast, protein content was significantly reduced in SPD compared to that in WB (from 15.62 ± 0.09 g/100 g to 10.36 ± 0.18 g/100 g). SPD and MEC showed a significant increase in free phosphorous values (19.17- and 12-fold increase, respectively) and a significant reduction in phytic acid content (3.31- and 1.89-fold decrease, respectively). Finally, TSPC and soluble FA isomers significantly increased in SPD (1.89- and 43.97-fold, respectively) and MEC (1.22- and 26.05-fold, respectively) with respect to WB.

### Characterization of Phenolic Compounds

The phenolic profile of GERM, SPD, and MEC was determined by high-performance liquid chromatography coupled with electrospray ionization quadrupole time-of-flight mass spectrometry in tandem (HPLC-ESI-QTOF-MS/MS) in negative ionization mode ([M - H]^−^) and classified according to peak elution, identification/tentative identification, molecular formula, retention time (RT), fragmentation score, mass error (in ppm), precursor, and product ions (*m/z*). Phenolic compounds were identified by comparison of reference standards, or tentatively identified by comparison of RT, fragmentation pattern, error between observed and theoretical mass, and molecular formulas with those reported in the literature for wheat. In GERM, 20 phenolic compounds were tentatively identified (Table 2). Using reference standards, three of the 11 phenolic acids were identified, as previously described by Gawlik-Dziki et al. (Gawlik-Dziki et al., 2016) in wheat extracts germinated at 20°C for 4 days: 4-hydroxybenzoic acid (i1) (1), caffeic acid (3) and *trans*-FA (12), respectively. Similar to compound 1, peak 17 with observed [M—H]^−^ at *m/z* 137 and RT at 22.8 min was tentatively characterized as isomer 2 of 4-hydroxybenzoic acid. Compounds 2 and 7 with theoretical [M—H]^−^ at *m/z* 371 and compound 4 with theoretical [M—H]^−^ at 367 and a daughter ion at *m/z* 193 (corresponding to a deprotonated FA), were identified as FA derivatives (i1 and i2) and 3-feruloylquinic acid, respectively (Barros Santos et al., 2019). Compounds 6, 10, and 13 with detected precursor ion [M—H]^−^ at *m/z* 385 and main fragment ions at *m/z* 205, 267, and 175, respectively, were tentatively characterized as 1-*O*-sinapoyl-beta-D-glucose (i1, i2, and i3, respectively) (Barros Santos et al., 2019). Regarding the flavonoid class, compound 9 with molecular formula C_26_H_28_O_15_ and [M—H]^−^ at *m/z* 579 and lacking fragment ions was tentatively identified as lucenin-1/3 (luteolin-6/8-C-xyloside-8/6-C-glucoside), in agreement with data previously reported by other authors in un-germinated wheat (Dinelli et al., 2009; Leoncini et al., 2012). Similarly, compound 18 with [M—H]^−^ at *m/z* 329 and compounds 19 and 20 with [M—H]^−^ at *m/z* 443 were assigned as 5,7,4′-trihydroxy-3′,5′-dimethoxy-flavone (tricin) and isomers 1 and 2 of formononetin (glycosylated and methylated), respectively (Dinelli et al., 2011). Peaks 11, 14, and 16, which showed the same precursor ion at *m/z* 563 [M—H]^−^ and gave main daughter ions at *m/z* 353, 443, and 383, were identified as isomers 1, 2, and 3 of apigenin-6-C-arabinoside-8-C-hexoside isomers (Dinelli et al., 2011). Finally, compound 5 with the molecular formula C_16_H_20_O_9_ and precursor ion at *m/z* 355.1029 [M—H]^−^, and compound 15 with molecular formula C_11_H_12_O_5_ and observed [M - H]^−^ at *m/z* 223.0622 were not able to be identified because of lack of a coincident fragmentation pattern in the literature.

**Table 2:** Tentative identification of GERM phenolic compounds by HPLC-ESI-QTOF-MS/MS in negative ion mode.

**No**	**Proposed compounds**	**Molecular formula**	**RT (min)**	**Score**	**Error (ppm)**	**Precursor ion (*m/z*)**	**MS/MS (relative intensity, %)**
1	4-hydroxybenzoic acid (i1)	C_7_H_6_O_3_	7.6	70.12	−5.42	137.0242	nd
2	Ferulic acid derivative (i1)	C_16_H_20_O_10_	9.6	73.94	−5.46	371.0999	75.0027 (9.90); 134.0363 (22.99); 193.0529 (100); 259.0270 (7.51); 371.1049 (9.58)
3	Caffeic acid	C_9_H_8_O_4_	10.8	65.52	−7.13	179.0369	nd
4	3–Feruloylquinic acid	C_17_H_20_O_9_	11.2	79.64	−7.58	367.1065	134.0364 (30.30); 193.0491 (100); 278.0699 (0.36); 367.1019 (1.70)
5	Unidentified	C_16_H_20_O_9_	12.9	75.67	−7.24	355.1029	101.0134 (6.55); 175.0446 (100); 268.9671 (0.99)
6	1–*O*–Sinapoyl-beta-D-glucose (i1)	C_17_H_22_O_10_	14	48.93	−7.34	385.1146	89.0247 (20.72); 149.019 (8.54); 205.0531 (100); 327.0984 (5.30)
7	Ferulic acid derivative (i2)	C_16_H_20_O_10_	16.4	81.86	−4.89	371.1002	121.0287 (74.52); 192.0509 (5.36); 249.0575 (100); 371.0996 (55.15)
8	Syringic acid	C_9_H_10_O_5_	16.5	85.32	−2.14	197.0455	nd
9	Lucenin-1/3 (luteolin-6/8-C-xyloside-8/6-C-glucoside)	C_26_H_28_O_15_	17.8	90.54	−2.9	579.1370	nd
10	1-*O*-Sinapoyl-beta-D-glucose (i2)	C_17_H_22_O_10_	18	85.98	−4.55	385.1146	113.0225 (41.91); 180.9091 (27.15); 267.0778 (100); 307.1036 (8.86); 385.1130 (21.92)
11	Apigenin-6-C-arabinoside-8-C-hexoside isomer (i1)	C_26_H_28_O_14_	18.8	92.4	−3.28	563.1406	119.0276 (1.22); 163.2720 (0.33); 265.0513 (1.39); 353.0729 (8.61); 443.1027 (7.72); 519.9548 (2.22); 563.1417 (100)
12	*Trans*-ferulic acid	C_10_H_10_O_4_	20	64.01	9.33	193.0484	nd
13	1-O-Sinapoyl-beta-D-glucose (i3)	C_17_H_22_O_10_	20.1	46.96	−6.73	385.1146	129.0205 (24.84); 175.0386 (100); 265.0693 (33.25); 353.0627 (7.5)
14	Apigenin-6-C-arabinoside-8-C–hexoside isomer (i2)	C_26_H_28_O_14_	20.6	83.84	−5.75	563.1406	117.6116 (0.17); 178.0311 (1.00); 233.0466 (0.48); 311.0466 (0.48); 383.0793 (5.21); 443.0973 (9.30); 563.1445 (100)
15	Unidentified	C_11_H_12_O_5_	20.7	94.28	−4.04	223.0622	59.0163 (29.91); 91.0523 (100); 117.0739 (26.17); 176.9861 (31.09); 223.0803 (3.50)
16	Apigenin-6-C-arabinoside-8-C-hexoside isomer (i3)	C_26_H_28_O_14_	22.5	61.45	−5.11	563.1440	nd
17	4-hydroxybenzoic acid (i2)	C_7_H_6_O_3_	22.8	47.11	−2.08	137.0247	nd
18	5,7,4'-trihydroxy-3',5'-dimethoxy-flavone (tricin)	C_17_H_14_O_7_	34.5	76.24	−7.79	329.0694	nd
19	Formononetin (Glycosylated and methylated) (i1)	C_23_H_24_O_9_	34.9	52.19	−9.38	443.1390	nd
20	Formononetin (Glycosylated and methylated) (i2)	C_23_H_24_O_9_	35.5	92.9	−3.15	443.1362	nd

*RT, retention time; i, isomer; nd, not detected*.

In both SPD and MEC, 14 phenolic compounds were identified (Table 3). 4-Hydroxybenzoic acid (i1) (3), vanillic acid (4), 2,4-dihydroxybenzoic acid (i3) (5), caffeic acid (6), vanillin (7), and *trans*-FA (10) were confirmed by reference standards. These phenolic acids have been previously reported by Martín-Diana et al., 2021 and Cengiz et al. (2021) in powdered WB hydrolysates. Moreover, additional peaks with molecular formula and *m/z* same as that of reference standards but different RT and MS spectra were tentatively identified as isomeric structures. Compounds 1 and 2(*m/z* 153[M—H]^−^) with RT of 4 and 5 min were characterized as isomer 1 and 2 of 2,4-dihydroxybenzoic acid, respectively. Compound 12 (*m/z* 193 [M—H]^−^) with RT of 21 min was identified as *cis*-FA, and compound 13 with theoretical [M—H]^−^ at *m/z* 137 and RT of 23 min was identified as 4-hydroxybenzoic acid (i2). Finally, MS/MS spectral analysis revealed the presence of peaks at 16, 19, 21, and 24 min, which presented the same father ion of *m/z* 563 [M—H]^−^ and produced a similar MS^2^ fragmentation pattern. These compounds (8, 9, 11, and 14) were tentatively characterized as apigenin-6-C-arabinoside-8-C-hexoside isomers (i1, i2, i3, and i4, respectively) (Dinelli et al., 2011).

**Table 3:** Tentative identification of SPD and MEC phenolic compounds by HPLC-ESI-QTOF-MS/MS in negative ion mode.

**No**	**Proposed compounds**	**Molecular formula**	**Ingredient**	**RT (min)**	**Score**	**Error (ppm)**	**Precursor ion (*m/z*)**	**MS/MS (relative intensity, %)**
1	2,4-dihydroxybenzoic acid (i1)	C_7_H_6_O_4_	SPD	4.3	79.72	−1.56	153.0193	82.9958 (100); 153.0128 (72.61)
			MEC	4.2	70.71	−6.02	153.0198	nd
2	2,4-dihydroxybenzoic acid (i2)	C_7_H_6_O_4_	SPD	5.2	80.71	−6.71	153.0203	53.0384 (19.17); 91.0125 (10.41); 109.0284 (100)
			MEC	5.2	65.7	−12.56	153.0208	nd
3	4-hydroxybenzoic acid (i1)	C_7_H_6_O_3_	SPD	7.7	81.68	−12.88	137.0263	65.0072 (13.63); 108.0191 (33.55); 137.0221 (100)
			MEC	7.7	84.01	−4.53	137.025	137.0221 (100)
4	Vanillic acid	C_8_H_8_O_4_	SPD	9.9	73.17	−7.33	167.0364	nd
			MEC	10.0	62.52	−5.11	167.0365	nd
5	2,4-dihydroxybenzoic acid (i3)	C_7_H_6_O_4_	SPD	10.7	99.14	−0.2	153.0194	65.0371 (34.28); 109.0259 (100); 153.0091 (23.74)
			MEC	10.7	72.47	−6.58	153.0199	nd
6	Caffeic acid	C_9_H_8_O_4_	SPD	11.3	56.04	−12.96	179.0367	93.0352 (22.58); 135.0432 (100)
		C_9_H_8_O_4_	MEC	11.3	64.89	−5.45	179.0353	nd
7	Vanillin	C_8_H_8_O_3_	SPD	15.1	74.7	−3.39	151.0408	nd
			MEC	15.1	65.53	−10.59	151.0414	nd
8	Apigenin-6-C-arabinoside-8-C-hexoside (i1)	C_26_H_28_O_14_	SPD	16.7	60.69	3.81	563.1431	210.8969 (4.49); 277.1295 (2.48); 325.0840 (3.25); 383.0773 (17.20); 415.2465 (10.63); 528.2741 (7.92); 563.1344 (100)
			MEC	16.6	67.22	−0.77	563.1397	nd
9	Apigenin-6-C-arabinoside-8-C-hexoside (i2)	C_26_H_28_O_14_	SPD	18.9	80.57	−5.2	563.1431	202.0162 (2.19); 260.5279 (0.36); 311.1749 (1.53); 353.0562 (8.48); 413.0764 (1.31); 443.1070 (11.44); 563.1475 (100)
			MEC	18.8	88.05	0.11	563.1407	nd
10	*Trans*-ferulic acid	C_10_H_10_O_4_	SPD	20.0	96.39	−3.95	193.0513	51.2765 (0.20); 106.0406 (10.73); 134.0377 (100); 178.0224 (14.22)
			MEC	20.0	93.27	−4.59	193.0517	90.1390 (4.93); 134.0316 (100); 167.0645 (19.17)
11	Apigenin−6–C–arabinoside-8-C-hexoside (i3)	C_26_H_28_O_14_	SPD	20.7	71.64	−7.11	563.1431	205.0360 (2.22); 259.1010 (0.84); 325.0530 (2.24); 383.0832 (13.22); 443.1033 (8.06); 527.2743 (2.66); 563.1449 (100)
			MEC	20.6	82.52	−5.81	563.1442	123.0069 (2.14); 197.8871 (2.20); 353.0616 (15.28); 383.0952 (8.47); 426.9918 (4.60); 503.1427 (4.89); 563.1469 (100)
12	*Cis*-ferulic acid	C_10_H_10_O_4_	SPD	21.7	80.41	−10.5	193.0528	89.0308 (23.47); 137.0334 (100); 179.0332 (6.09)
			MEC	21.7	80.41	−10.4	193.0528	63.0035 (47.37); 133.0224 (43.97)
13	4–hydroxybenzoic acid (i2)	C_7_H_6_O_3_	SPD	23.0	81.68	−12.88	137.0263	nd
			MEC	22.8	84.01	−4.53	137.0250	nd
14	Apigenin-6-C-arabinoside-8-C-hexoside (i4)	C_26_H_28_O_14_	SPD	23.5	59.91	−5.18	563.1431	263.0646 (6.96); 383.0582 (12.52); 443.1119 (7.22); 503.1066 (2.86); 563.1420 (100)
			MEC	23.4	44.05	−8.29	563.1444	nd

*SPD, hydrolyzed WB spray-dried at 130°C; MEC, hydrolyzed WB spray-dried at 130°C and microencapsulated with Pisane C_9_; i, isomer; nd, not detected; RT, retention time*.

In order to clarify the phenolic profile of soluble WB powders, the total relative content of phenolic compounds (TRCP) was calculated from the sum of all phenolic compounds quantified by chromatography (mg/100 g d.w.) (Table 4). TRCP in GERM was 55.24 ± 0.95 mg/100 g, of which hydroxycinnamic acid derivatives were the predominant phenolic class (74.76% of TRCP), with FA derivative isomer 1 being the compound with major contribution (25.63% of TRCP). Smaller amounts of flavonoids (25.24% of TRCP) composed of flavones (13.06% of TRCP) and isoflavonoids (12.18% of TRCP) were observed.

**Table 4:** Quantification and bioaccessibility percentage of the phenolic compounds of GERM.

**Phenolic compounds**	**Non-digested**	**Gastric phase**	**% Bioaccessibility**	**Intestinal phase**	**Bioaccessibility (%)^**9**^**
**TSPC** ^**1**^	0.43 ± 0.01^a^	0.56 ± 0.02^b^	132.19	0.61 ± 0.04^c^	141.69
4-hydroxybenzoic acid (i1)^2^	tr	tr	–	tr	–
4-hydroxybenzoic acid (i2)^2^	tr	tr	–	tr	–
Syringic acid^3^	tr	tr	–	tr	–
**Total hydroxybenzoic acids**	tr	tr	–	tr	–
*Trans*-ferulic acid^4^	1.27 ± 0.41^a^	3.34 ± 0.29^b^	215.71	4.61 ± 0.17^c^	300.52
Caffeic acid^5^	tr	tr	–	tr	–
Ferulic acid derivative (i1)^4^	14.16 ± 0.76^b^	15.34 ± 0.51^b^	108.62	5.11 ± 0.16^a^	36.15
Ferulic acid derivative (i2)^4^	5.35 ± 0.11^a^	9.73 ± 3.64^a^	182.69	5.60 ± 0.16^a^	104.78
3-Feruloyl quinic acid^4^	4.89 ± 0.21^b^	4.74 ± 1.20^b^	97.44	0.56 ± 0.04^a^	11.43
1*-O-*Sinapoyl-β-D-glucose (i1)^4^	11.25 ± 0.11	nd	–	nd	–
1*-O-*Sinapoyl-β-D-glucose (i2)^4^	3.87 ± 0.18	nd	–	nd	–
1*-O-*Sinapoyl-β-D-glucose (i3)^4^	0.52 ± 0.06	nd	–	nd	–
**Total hydroxycinnamic acids**	41.30 ± 1.20^b^	33.15 ± 5.35^b^	80.48	15.87 ± 0.36^a^	38.46
Apigenin-6-C-arabinoside-8-C-hexoside (i1)^6^	1.69 ± 0.02^c^	0.89 ± 0.02^a^	52.97	1.07 ± 0.02^b^	63.19
Apigenin-6-C-arabinoside-8-C-hexoside (i2)^6^	5.52 ± 0.08^b^	4.59 ± 0.44^a^	82.97	4.61 ± 0.12^a^	83.41
Apigenin-6-C-arabinoside-8-C-hexoside (i3)^6^	tr	tr	–	tr	–
5,7,4'-trihydroxy-3',5'-dimethoxy-flavone (tricin)^7^	tr	tr	–	tr	–
Lucenin-1/3 (luteolin-6/8-C-xyloside-8/6-C-glucoside)^6^	tr	tr	–	tr	–
**Total flavones**	7.21 ± 0.10^b^	5.48 ± 0.46^a^	75.95	5.67 ± 0.15^a^	78.68
Formononetin (Glycosylated and methylated) (i1)^8^	1.51 ± 0.12	nd	–	nd	–
Formononetin (Glycosylated and methylated) (i2)^8^	5.22 ± 0.04	nd	–	nd	–
**Total isoflavonoids**	6.73 ± 0.16	nd	–	nd	–

*Data are mean values ± standard deviation of three replicates. Different lowercase letters in the same row indicate significant differences of means in different digestion phases (one-way ANOVA, post-hoc Duncan's test, p ≤ 0.05). i, isomer; nd, non-detected; tr, traces; TSPC, total soluble phenolic compounds. ^1^g gallic acid equivalents/100 g. ^2^mg 100/g 4-hydroxybenzoic acid (linearity range 0–3.75 μg/mL, R^2^ > 0.99). ^3^mg/100 g gallic acid (linearity range 0.1–8.33 μg/mL, R^2^ > 0.99). ^4^mg/100 g ferulic acid (linearity range 0–25 μg/mL, R^2^ = 1, LOD = 0.04 μg mL, LOQ = 0.12 μg/mL). ^5^mg/100 g caffeic acid (linearity range 0–2.77 μg/mL, R^2^ > 0.99, LOD = 0.16 μg/mL, LOQ = 0.47 μg/mL). ^6^mg/100 g vicenin-2 (linearity range 0–25 μg/mL, R^2^ = 1, LOD = 0.02 μg/mL, LOQ = 0.05 μg/mL). ^7^mg/100 g vitexin (linearity range 0–25 μg/mL, R^2^ = 1, LOD = 0.22 μg/mL, LOQ = 0.67 μg/mL). ^8^mg/100 g daidzein (linearity range 0–8.33 μg/mL, R^2^ = 1, LOD = 0.03 μg/mL, LOQ = 0.09 μg/mL). ^9^Bioaccessibility expressed as percentage was calculated as the ratio of free phenolic compounds released from food matrix during digestion and the initial content of free phenolic compounds in the wheat ingredients before digestion (values over 100% are indicative of increase in the bioaccessible amount of phenolic compounds in each digestion phase)*.

The phenolic compound contents of SPD and MEC are presented in Table 5. In SPD, a higher content of TSPC and TRCP (1.55- and 1.6-fold, respectively) was detected than in MEC. The most abundant phenolic subclass was hydroxycinnamic acids in both SPD and MEC (90.37 and 85.57% of TRCP, respectively) followed by smaller amounts of flavones, hydroxybenzoic acids, and hydroxybenzaldehydes, with *trans*-FA being the main phenolic acid in both SPD and MEC (175.22 ± 3.26 mg/100 g and 102.7 ± 3.26 mg/100 g, respectively). Compared to SPD, a significantly lower value was observed in *trans*-FA (1.70-fold) and isomers 2 and 3 of apigenin diglucosides in MEC (1.52-, and 1.48-fold, respectively). Vanillic acid and vanillin showed significantly higher values in MEC than in SPD (1.36- and 3.25-fold increase), although nonsignificant differences were detected in 4-hydroxybenzoic acid (i1) and *cis*-FA.

**Table 5:** Quantification and bioaccessibility percentage of the phenolic compounds of SPD and MEC.

**Phenolic compounds**	**SPD**					**MEC**				
	**Non-digested**	**Gastric**	**Bioaccessibility**	**Intestinal**	**Bioaccessibility**	**Non–digested**	**Gastric**	**Bioaccessibility**	**Intestinal**	**Bioaccessibility**
		**phase**	**(%) ^**9**^**	**phase**	**(%)**		**phase**	**(%)**	**phase**	**(%)**
**TSPC** ^1^	0.93 ± 0.02^b^	0.86 ± 0.02^a^	93.03	0.84 ± 0.02^a^	90.47	0.60 ± 0.01^b^^*^	0.50 ± 0.01^a^^*^	84.10	0.72 ± 0.01^c^^*^	120.14
2,4-dihydroxybenzoic acid (i1)^2^	tr	tr	–	tr	–	tr	tr	–	tr	–
2,4-dihydroxybenzoic acid (i2)^2^	tr	tr	–	tr	–	tr	tr	–	tr	–
2,4-dihydroxybenzoic acid (i3)^2^	tr	tr	–	tr	–	tr	tr	–	tr	–
4-hydroxybenzoic acid (i1)^3^	4.35 ± 0.18 ^b^	5.93 ± 0.17 ^c^	136.52	2.24 ± 0.35 ^a^	51.51	4.71 ± 0.07 ^b^	1.31 ± 0.12 ^a^,^*^	27.97	tr	–
4-hydroxybenzoic acid (i2)^3^	tr	tr	–	tr	–	tr	tr	–	tr	–
Vanillic acid^4^	4.33 ± 0.07^b^	6.27 ± 0.42^c^	144.93	2.59 ± 0.07^a^	59.82	5.87 ± 0.14^b^^*^	0.74 ± 0.09^a^,^*^	12.65	nd	–
**Total hydroxybenzoic acids**	8.68 ± 0.12^b^	12.21 ± 0.60^c^	140.62	4.83 ± 0.41^a^	55.68	10.57 ± 0.21^b^^*^	2.06 ± 0.21^a^^*^	19.47	tr	–
Caffeic acid^5^	tr	tr	–	tr	–	tr	tr	–	tr	–
*Trans*-ferulic acid^6^	175.22 ± 3.26^a^	227.06 ± 9.13^b^	129.66	178.10 ± 1.60^a^	101.68	102.70 ± 2.79^c^^*^	91.15 ± 4.63^b^^*^	88.81	78.17 ± 5.90^a^^*^	76.64
*Cis*-ferulic acid^6^	3.09 ± 0.08^a^	4.96 ± 0.34^b^	160.81	5.09 ± 0.33^b^	164.94	2.28 ± 0.30^b^	1.61 ± 0.12^a^^*^	70.91	1.63 ± 0.01^a^^*^	72.09
**Total hydroxycinnamic acids**	178.31 ± 0.08^a^	232.02 ± 0.34^c^	130.12	183.19 ± 0.33^b^	102.74	104.98 ± 0.30^c^^*^	92.76 ± 0.12^b^^*^	88.36	79.80 ± 0.01^a^^*^	76.01
Apigenin-6-C-arabinoside-8-C-hexoside(i1)^7^	tr	tr	–	tr	–	tr	nd	–	nd	–
Apigenin-6-C-arabinoside-8-C-hexoside(i2)^7^	3.12 ± 0.18^a^	4.02 ± 0.06^b^	129.23	3.06 ± 0.07^a^	98.32	2.05 ± 0.01^*^	tr	–	tr	–
Apigenin-6-C-arabinoside-8-C-hexoside(i3)^7^	7.12 ± 0.58^a^	9.15 ± 0.02^b^	128.88	8.19 ± 0.37^ab^	115.13	4.81 ± 0.05^c^^*^	0.31 ± 0.11^a^^*^	6.40	1.01 ± 0.07^b^^*^	21.09
Apigenin-6-C-arabinoside-8-C-hexoside(i4)^7^	tr	tr	-	tr	–	tr	tr	–	tr	–
**Total flavones**	10.24 ± 0.75^a^	13.17 ± 0.07^b^	128.97	11.25 ± 0.45^a^	110.01	6.86 ± 0.04^c^^*^	0.31 ± 0.11^a^^*^	4.49	1.01 ± 0.07^b^^*^	14.79
Vanillin^8^	0.08 ± 0.01^a^	0.44 ± 0.01^c^	583.65	0.33 ± 0.06^b^	424.35	0.26 ± 0.02^*^	tr	–	tr	–
**Total hydroxybenzaldehydes**	0.08 ± 0.01^a^	0.44 ± 0.01^c^	583.65	0.33 ± 0.06^b^	424.35	0.26 ± 0.02^*^	tr	–	tr	–

*Data are mean values ± standard deviation of three replicates. Different lowercase letters in the same row indicate significant differences between means in different digestion phases (one-way ANOVA, post-hoc Duncan's test, p ≤ 0.05)*.

* *denotes significant differences among wheat derived ingredients in the same phases of digestion (one-way ANOVA, post-hoc Duncan's test, p ≤ 0.05). SPD, hydrolyzed WB spray-dried at 130°C; MEC, hydrolyzed WB spray-dried at 130°C and microencapsulated with Pisane C_9_; i, isomer; nd, non-detected; TSPC, total soluble phenolic compounds; tr, traces. ^1^g/100 g gallic acid equivalents. ^2^mg/100 g 2,4-dihydroxybenzoic acid (linearity range 0–3.75 μg/mL, R^2^ = 1). ^3^mg/100 g 4-hydroxybenzoic acid (linearity range 0–3.75 μg/mL, R^2^ > 0.99). ^4^mg/100 g vanillic acid (linearity range 0.47–3.75 μg/mL, R^2^ > 0.98). ^5^mg/100 g caffeic acid (linearity range 0–2.77 μg mL, R^2^ > 0.99, LOD = 0.16 μg mL, LOQ = 0.47 μg/mL). ^6^mg/100 g ferulic acid (linearity range 0–25 μg/mL, R^2^ = 1, LOD = 0.04 μg/mL, LOQ = 0.12 μg/mL). ^7^mg/100 g vicenin-2 (linearity range 0–25 μg/mL, R^2^ = 1, R^2^ = 1, LOD = 0.02 μg/mL, LOQ = 0.05 μg/mL). ^8^mg/100 g vanillin (linearity range 0–3.75 μg/mL, R^2^ = 1). ^9^Bioaccessibility expressed as percentage was calculated as the ratio of free phenolic compounds released from food matrix during digestion and the initial content of free phenolic compounds in the wheat ingredients before digestion (values over 100% are indicative of increase in the bioaccessible amount of phenolic compounds in each digestion phase)*.

### Bioaccessibility of Phenolic Compounds in Different Digestion Stages

The bioaccessibility of TSPC significantly increased after gastric and intestinal digestion of GERM (32.19 and 41.69% increase, respectively; Table 4). FA derivative (i1) was the most abundant compound (15.34 mg/100 g) observed in the gastric step and FA derivative (i2) (5.6 mg/100 g) in the intestinal phase. *Trans*-FA concentration gradually increased in both digestion phases (2.63- and 3.63-fold increase, respectively). The gastric phase did not affect the bioaccessibility of FA derivatives (i1 and i2) and 3-feruloyl quinic acid in GERM. However, isomer 1 of FA derivative and 3-feruloyl quinic acid significantly decreased their bioaccessibility (36.15 and 11.43%, respectively), and isomer 2 remained unaltered (104.78% bioaccessibility) in the intestinal phase. Gastric and intestinal conditions significantly affected apigenin isomers (i1 and i2), decreasing their bioaccessibility (up to 47.03 and 17.03%, respectively), and 1-*O*-sinapoyl-β-D-glucose isomers (i1, i2, and i3) were not detected in both phases. These changes were reflected by bioaccessibility reduction in hydroxycinnamic acids and flavones in the gastric (by 19.52 and 24.05%, respectively) and intestinal (by 61.54 and 21.32%, respectively) phases. Isoflavones were not detected after simulated digestion.

Regarding SPD, a slight reduction in TSPC bioaccessibility was observed with no significant differences between the gastric and intestinal phases (93.03 and 90.47%, respectively; Table 5). The major compound in both gastric and intestinal phases was *trans*-FA (227.06 ± 9.13 and 178.1 ± 1.6 mg/100 g, respectively). The bioaccessibility of hydroxybenzoic (4-hydroxybezoic acid and vanillic acid) and hydroxycinnamic (*trans*- and *cis*-FA) acids, flavones (apigenin diglucoside i2 and i3), and hydroxybenzaldehydes (vanillin) was favored by gastric conditions, increasing by 40.62, 30.12, 28.97, and 483.65%, respectively. However, a significant reduction of bioaccessibility was observed after the intestinal phase in each phenolic class, with hydroxybenzoic acid bioaccessibility the only one with values lower than 100% (55.68% bioaccessibility). *Cis*-FA and isomer 3 of apigenin diglucoside maintained their bioaccessibility after intestinal digestion.

Regarding bioaccessibility in MEC, TSPC increased under intestinal conditions (36.04%) compared to gastric phase (84.10%), with intestinal bioaccessibility values being significantly higher than those reported for SPD (1.33-fold increase; Table 5). Like in SPD gastric and intestinal phenolic fractions, *trans*-FA was the main compound in MEC fractions (102 ± 2.79 and 78.17 ± 5.9 mg/100 g, respectively). In addition, a similar composition was observed in the gastric phase of MEC compared to that of SPD, except for apigenin-diglucoside i2 and vanillin, which were not quantifiable after simulated digestion, and apigenin diglucoside (i1), which was not detected. These modifications were reflected by decrease in hydroxybenzoic and hydroxycinnamic acid, and flavone bioaccessibility, which was significantly lower in MEC than in SPD (7.22-, 1.47-, and 28.72-fold, respectively). In the intestinal step, hydroxybenzoic acids were not quantifiable, hydroxycinnamic acids decreased by 12.35%, and flavones slightly increased (3.29-fold increase) compared to the gastric phase in MEC. As MEC was composed of Pisane C_9_ in a ratio of 1:1, TSPC pea protein was determined (0.2 ± 0.01 g/100 g). After digestion, TSPC Pisane C_9_ showed a significant decrease in the bioaccessibility of 30% in both the gastric and intestinal phases (Supplementary Figure 1).

### Effect of *in vitro* Digestion on TAC

Total antioxidant capacity (TAC) was measured in germinated (GERM) and WB hydrolysates (SPD and MEC) in order to evaluate the effect of the digestive process on TAC through different methods (DPPH, ABTS^+^, ORAC, and FRAP) (Table 6).

**Table 6:** Effect of *in vitro* digestion on antioxidant activity of wheat ingredients.

**Ingredient**	**Digestion phase**	**DPPH (μmol TE/100 g)**	**ABTS (μmol/TE 100 g)**	**ORAC (μmol TE/100 g)**	**FRAP (mmol Fe^**+2**^/100 g)**
GERM	Non-digested	1133.96 ± 118.04^b, A^	3434.28 ± 58.38^b, A^	4080.59 ± 247.12^b, A^	1845.72 ± 58.65^b, A^
	Gastric	1249.15 ± 47.68^b, A^	4631.08 ± 400.93^a, B^	12216.26 ± 747.32^b, B^	2696.24 ± 20.39^b, B^
	Intestinal	3451.72 ± 97.77^c, B^	16642.24 ± 670.75^a, C^	22623.05 ± 1074.10^b, C^	5320.77 ± 200.02^b, C^
SPD	Non-digested	2724.59 ± 13.52^d, B^	11519.50 ± 551.18^d, B^	12896.99 ± 606.95^d, A^	5647.30 ± 133.62^d, A^
	Gastric	1696.99 ± 136.91^c, A^	9203.99 ± 477.61^b, A^	15109.47 ± 1341.92^b, B^	6063.72 ± 51.40^d, B^
	Intestinal	3235.13 ± 1279.13^c, B^	23183.00 ± 2498.27^b, C^	20456.35 ± 1103.22^b, C^	9386.18 ± 451.83^d, C^
MEC	Non-digested	1508.98 ± 9.67^c, B^	7224.50 ± 290.68^c, A^	7369.20 ± 387.94^c, A^	2898.20 ± 3.98^c, A^
	Gastric	1178.26 ± 70.53^b, A^	13299.55 ± 188.80^d, B^	12429.18 ± 2150.54^b, B^	3329.17 ± 76.54^c, B^
	Intestinal	1577.07 ± 110.20^b, B^	27961.34 ± 988.97^c, C^	20546.94 ± 941.81^b, C^	6640.79 ± 335.46^c, C^
Pisane C_9_	Non-digested	70.73 ± 6.53^a, A^	2173.08 ± 128.96^a, A^	1339.03 ± 101.55^a, A^	498.51 ± 27.52^a, A^
	Gastric	205.99 ± 29.67^a, B^	12719.55 ± 282.45^c, B^	9221.86 ± 1833.35^a, B^	891.02 ± 97.22^a, B^
	Intestinal	278.71 ± 23.04^a, C^	37187.78 ± 1757.47^d, C^	19665.77 ± 712.94^a, C^	473.02 ± 11.02^a, A^

*Data are mean values ± standard deviation of three replicates. Different lowercase letters in the same row indicate significant differences among digestion phases (one-way ANOVA, post-hoc Duncan's test, p ≤ 0.05). Different uppercase letters denote significant differences among ingredients in the same phase of digestion (one-way ANOVA, post-hoc Duncan's test, p ≤ 0.05). ABTS, 2,2'- azino-bis (3-ethylbenzothiazoline-6-sulfonic acid); DPPH, 2,2-diphenyl-1-picryl-hydrazyl-hydrate; FRAP, ferric reducing antioxidant power; GERM, germinated whole-wheat grain; SPD, hydrolyzed WB spray-dried at 130°C; MEC, hydrolyzed WB spray-dried at 130°C and microencapsulated with Pisane C_9_ (MEC); ORAC, oxygen radical absorbance capacity*.

GERM showed the lowest (*p* ≤ 0.05) antioxidant activity, as determined by the four methods, compared to SPD and MEC (Table 6). Undigested SPD showed a significantly (*p* ≤ 0.05) higher antioxidant properties than MEC. Pisane C_9_ was also evaluated in order to know its contribution to the antioxidant MEC properties, since it was used for encapsulation. Pisane C_9_ resulted in a very low antioxidant activity, as compared with SPD and MEC. After gastric digestion (Table 6), GERM antioxidant activity increased between 10 and 40%, as determined by DPPH, ABTS^+^, and FRAP assays, whereas ORAC values increased 3-fold. The antioxidant activity of SPD gastric digests was slightly reduced by the DPPH and ABTS^+^ methods, whereas a significant (*p* ≤ 0.05) increase was observed for ORAC and FRAP values. Similarly, the antioxidant activity of MEC increased after the gastric phase, as indicated by ABTS^+^, ORAC and FRAP values. This increase was higher than the one observed in the case of the SPD, because of the contribution of Pisane C_9_, which enhanced its activity between 2-and 6-fold, depending on the *in vitro* assay method. After intestinal digestion (Table 6), all the ingredients significantly (*p* ≤ 0.05) increased its TAC with respect to the gastric phase, regardless of the assay used for the analysis (DPPH, ABTS^+^, ORAC, or FRAP). At the end of the intestinal phase of digestion, SPD showed better antioxidant profile than GERM and MEC based on the analysis of DPPH, ORAC, and FRAP values.

### Effect of *in vitro* Digestion on Anti-inflammatory Activity (AIA)

In this study, we investigated whether the anti-inflammatory potential of wheat nutraceutical ingredients (GERM, SPD, and MEC) was affected by physiological conditions during the gastric and intestinal phases of digestion. First, cell viability was measured after the exposure of RAW264.7 cells to different doses (0.05–0.5 mg/m) of gastric and intestinal digests for 20 h. No cytotoxic effects were observed between cells treated at the highest dose of extracts (0.5 mg/m) and negative control (non-treated cells) (data not shown). The positive control cells (exposed to LPS for 20 h) showed increased levels of IL-6 from undetected to 2.1 ng/ml and TNF-α from 0.1 to 8.4 pg/ml of growth media (data not shown). To evaluate the inhibitory effects of functional wheat ingredients on inflammation, RAW264.7 cells were pre-exposed for 1 h to the soluble fraction of powdered samples at three doses (0.05, 0.1, and 0.5 mg/ml), before stimulation with LPS for 20 h (40 ng/mL). Before *in vitro* digestion, GERM, SPD, and MEC extracts showed a significant reduction in cytokine release regardless of the dose of treatment (34.1–75.63% inhibition for IL-6 and from 24.16 to 56.31% inhibition for TNF-α) (Table 7). In general, a notable reduction in IL-6 and TNF-α values was observed in cells treated with non-digested GERM and MEC as compared to SPD extracts, with the lowest doses (0.05 mg/mL) of GERM (75.63% inhibition for IL-6) and MEC (55.05% inhibition for TNF-α) being the most effective treatments. Furthermore, a higher dose of SPD (0.5 mg/ml) was needed to reach the same reduction of IL-6 values as GERM (0.05 mg/mL) (*p* ≤ 0.05). Concerning non-digested extracts, a significant improvement in the reduction of both IL-6 and TNF-α (by about 90%) was detected when the cells were treated with the intestinal ingredient extract at a dose of 0.5 mg/ml. Functional ingredients did not increase AIA against IL-6 under digestion conditions. In the same way, the ability to reduce TNF-α concentration remained stable in cells treated with GERM and was negatively affected in those treated with MEC after gastric conditions. However, SPD significantly increased the inhibition of the latter cytokine after the gastric phase of digestion. Overall, there were no significant differences in TNF-α inhibition between the gastric and intestinal conditions. Pisane C_9_ did not show any anti-inflammatory activity in IL-6 and TNF-α secretion before and after simulated digestion at studied doses.

**Table 7:** Effect of *in vitro* digestion on the anti-inflammatory activity of wheat ingredients.

**Ingredient**	**Digestion phase**	**Dose (mg/mL)**					
		**0.05**	**0.1**	**0.5**	**0.05**	**0.1**	**0.5**
		**IL-6 (% inhibition)**			**TNF-α** **(% inhibition)**		
GERM	Non-digested	75.63 ± 0.00^d, B^	56.56 ± 4.08^c, A^	65.12 ± 3.26^b, A^	39.34 ± 7.57^ab, A^	38.30 ± 5.09^a, A^	48.95 ± 7.03^c, A^
	Gastric	76.36 ± 6.36^d, B^	61.71 ± 4.51^c, A^	51.59 ± 0.18^a, A^	35.69 ± 8.81^ab, A^	42.28 ± 3.14^abc, A^	40.40 ± 2.34^bc, A^
	Intestinal	63.96 ± 17.33^cd, A^	52.63 ± 0.73^bc, A^	100.00 ± 0.00^d, B^	37.63 ± 8.93^ab, A^	49.11 ± 4.41^bc, A^	97.06 ± 0.45^d, B^
SPD	Non-digested	34.94 ± 4.87^a, A^	34.18 ± 2.38^a, A^	75.01 ± 5.37^c, B^	29.47 ± 6.05^a, AB^	37.77 ± 3.21^a, B^	24.16 ± 9.42^a, A^
	Gastric	24.97 ± 1.29^a, A^	36.47 ± 9.05^a, A^	67.36 ± 4.73^bc, B^	57.32 ± 1.08^c, C^	51.75 ± 0.10^c, B^	46.60 ± 1.67^c, A^
	Intestinal	24.27 ± 2.57^a, A^	43.14 ± 5.35^ab, B^	99.70 ± 0.08^d, C^	45.34 ± 4.01^bc, A^	49.31 ± 6.20^bc, A^	92.43 ± 1.10^d, B^
MEC	Non-digested	64.81 ± 1.76^cd, B^	57.95 ± 0.88^c, A^	62.00 ± 0.98^b, AB^	55.05 ± 3.17^c, A^	56.31 ± 3.19^c, A^	43.55 ± 0.56^bc, A^
	Gastric	57.88 ± 0.81^bc, A^	63.58 ± 0.68^c, A^	65.94 ± 7.63^b, A^	34.40 ± 9.77^ab, A^	37.32 ± 4.09^a, A^	34.60 ± 4.20^b, A^
	Intestinal	51.02 ± 7.15^b, A^	38.47 ± 2.71^a, A^	99.93 ± 0.01^d, B^	41.89 ± 2.76^ab, A^	39.74 ± 3.26^ab, A^	96.40 ± 0.48^d, B^
		**0.025**	**0.05**	**0.25**	**0.025**	**0.05**	**0.25**
Pisane C_9_	Non-digested	nd	nd	nd	nd	nd	nd
	Gastric	nd	nd	nd	nd	nd	nd
	Intestinal	nd	nd	nd	nd	nd	nd

*Different uppercase letters in the same row indicate significant differences (p ≤ 0.05, Duncan's test) among doses (one-way ANOVA, post-hoc Duncan's test, p ≤ 0.05). Different lowercase letters in the same column denote significant differences among ingredients in the same phase of digestion (one-way ANOVA, post-hoc Duncan's test, p ≤ 0.05). GERM, germinated whole-wheat grain; SPD, hydrolyzed WB spray-dried at 130°C; MEC, hydrolyzed WB spray-dried at 130°C and microencapsulated with Pisane C_9_; IL, interleukin; TNF-α, tumor necrosis factor-alpha. Bold values are Dose (mg/mL) used for cell treatment with Pisane*.

## Discussion

Many diet-related diseases include oxidative damage as a starting point or a promoter in disease progression (Chatterjee, 2016). Excessive production of radical oxygen/nitrogen species resulting in synthesis and secretion of pro-inflammatory cytokines is a condition known as “low-grade chronic inflammation” that is also associated with several pre-pathological conditions, such as obesity and degenerative diseases (Williams et al., 2019). In this context, the development of functional foods containing nutrients capable of nurturing and optimizing humans' natural antioxidant and anti-inflammatory machinery has become a rather appealing concept (Mauro and Ilaria, 2016). In this field, whole grains and derived components (brans) have emerged as promising functional ingredient sources (Aune et al., 2016; Aborus et al., 2018; Cãlinoiu and Vodnar, 2018).

To enlarge the applications of WWG and WB as functional ingredients in foodstuffs that can promote human health, researchers have explored different technological treatments to improve the bioaccessibility of phenolic compounds from these food matrices and, subsequently, their health effects (Lemmens et al., 2019; Deroover et al., 2020; Onipe et al., 2021). From this perspective, the potential of WWG germination and WB transformation by hybrid processing routes (thermomechanical and enzymatic treatments) in solubilizing phenolic compounds and improving the antioxidant and anti-inflammatory properties of both raw materials has been highlighted in our recent studies (Bautista-Exposito et al., 2020; Tome-Sanchez et al., 2020; Martín-Diana et al., 2021). In this study, to clarify the effects of bioprocessing on WWG and WB, the overall nutritional and nutraceutical composition of GERM, SPD, and MEC was defined. All the bioactive compounds, to exert their functionality, must be stable to physiological conditions and bioaccessible for their absorption in the gastrointestinal tract (Ketnawa et al., 2021). For this reason, the release of phenolic compounds and change in their bioactivities were examined by *in vitro* simulated gastrointestinal digestion, foreseeing their use as functional food ingredients.

### GERM Had Increased Amounts of TDF, IDF, Monosaccharides, Free Phosphorous and Phenolic Compounds

Germination of WWG for 7 days at 21°C had profound effects on the carbohydrate fraction that include hydrolysis of poly/oligosaccharides, as indicated by the reduced content of starch, β-glucan, raffinose, stachyose, and sucrose, and accumulation of mono- and disaccharides (glucose > fructose > cellobiose > arabinose > galactose, Table 1). After seed imbibition, sprouting activates a wide spectrum of carbohydrolases, causing breakdown of starch, cellulose, glucomannans, arabinogalactans, arabinoxylans, raffinose family oligosaccharides, fructo-oligosaccharides, and sucrose, and making monosaccharides available (Benincasa et al., 2019). For instance, starch is partially hydrolyzed to glucose, maltose, and maltotriose by α-amylase and α-glucosidase. According to the literature, starch reduction may vary from 5 to 80% (in line with our results, Table 1) as function of the type of cereal grains (in terms of species, genotypes, varieties) and germination conditions (Lemmens et al., 2019). This is advantageous from a nutritional perspective, as the starch in sprouted cereals is generally more digestible because of the enzymatically modified structure of starch granules, thin cell walls, and readily available mono- and disaccharides (Lemmens et al., 2019).

Similarly, polysaccharides (present in cell walls) and oligosaccharides are hydrolyzed during germination by *de novo* synthesized enzymes producing changes in the composition of SDF and IDF of whole cereal grains that will depend on the type of cereal and germination conditions (Lemmens et al., 2019). Although noticeable reductions in β-glucans and raffinose family oligosaccharides were observed in GERM compared with WWG, SDF remained unchanged probably because of the formation of new tri- and tetrasaccharides from the depolymerization of celluloses and hemicelluloses, as indicated by the LC-MS/MS analysis (Supplementary Material 1). Higher content of insoluble polysaccharides contributing to the increased TDF content in GERM was an indicator of the biosynthesis of new cell walls from DF hydrolysis products. In agreement with our results, an earlier investigation reported an increased total and insoluble arabinoxylans in wheat shoots after 4 days of germination due to its utilization in the formation of cell walls of sprouts (Van Campenhout et al., 2007). Consumption of GERM may contribute to increased IDF intake, which is associated to enhanced gastrointestinal health by decreasing gut transit time and improving stool bulk (Cheng et al., 2021).

Germination did not affect the total protein content of WWG (as determined by the elemental analysis of nitrogen, Table 1). Previous studies have consistently shown that relative differences between cereal grains and sprouts are generally <10%, indicating that germination does not substantially affect total protein content (Lemmens et al., 2019). Nevertheless, it is important to mention that studies performed on wheat have shown that long germination times (3–7 days) at 20–25°C lead to partial hydrolysis of storage proteins by the action of seed endogenous proteases and peptidases, and accumulation of free amino acids (from 5 to 10 times) (Boukid et al., 2017; Marti et al., 2017). These changes in nitrogen fraction are usually associated to the improved protein digestibility (1.2–2-fold increase) of sprouted grains (Lemmens et al., 2019).

Majority of the phosphorous (P) in the WWG is stored as phytic acid that is mainly located in the aleurone layer of the bran (Deroover et al., 2020). Phytic acid is considered an anti-nutrient because of its ability to chelate divalent cations reducing the bioavailability of minerals (Deroover et al., 2020). In this study, the content of phytic acid was significantly reduced, increasing concomitantly free P content (Table 1). These effects were likely due to the action of phytases activated during germination (Cardone et al., 2020). Dephytinization induced by germination may oscillate between 6 and 23% (Lemmens et al., 2019), which is consistent with our findings, suggesting an improved mineral bioaccessibility of GERM with respect to WWG (Cardone et al., 2020).

Regarding the phenolic fraction of WWG, germination improved the content of soluble phenolic compounds, in line with the literature (Lemmens et al., 2019). Germination activates cell wall-degrading enzymes that include cellulases, hemicellulases, cinnamoyl esterases, and feruloyl esterases, whose combined action favors the release of bound phenolic compounds. Likewise, biosynthesis of phenolic compounds through the shikimate pathway could also explain the increased amounts of TSPC observed in GERM. The TSPC content found in GERM (0.43 ± 0.01 g GAE/100 g) was lower than the reported values by Aborus et al. (2018) (0.61 ± 0.05 g GAE/100 g) in wheat (*Triticum aestivum* L. ssp. *vulgare*) sprouted for 7 days at 17–21°C. These differences could be ascribed to genetic and environmental factors as well as the methodology used for TSPC analysis (Mpofu et al., 2006). Unlike Folin–Ciocalteu reaction, the FBBB reaction used in this study prevents overestimation in the amount of TSPC because of interferences with reducing sugars and enediols (Pico et al., 2020). The soluble phenolic fraction of GERM was mainly composed of hydroxycinnamic acids and minor amounts of hydroxybenzoic acids and flavonoids (Tables 2, 4), in agreement with earlier studies on different sprouted wheat varieties (Aborus et al., 2018a). The major phenolic compounds of GERM by order of abundance were FA derivative (i1) > apigenin diglucosides (i2) > FA derivative (i2) > formononetin (i2) > 3-feruloyl quinic acid. These phenolic compositions varied in comparison with the previous report by Aborus et al. (2018) on two varieties of wheat sprouted at 17–21°C for 7 days, where the dominant phenolic compounds determined were vanillic, protocatechuic, syringic, gallic, and sinapic acids. These differences might be attributed to genotype and growing environmental conditions (Pérez-Balibrea et al., 2011).

### SPD and MEC Had Improved Content of SDF, Monosaccharides, Free Phosphorous, and Phenolic Compounds, and Distinct Protein Content

To understand the changes in chemical composition of SPD as compared to WB is important to take into consideration all steps included in the processing route applied. As described previously, WB was submitted to the following cascade of treatments: autoclaving [121°C, 15 min], enzymatichydrolysis with Ultraflo XL [1% enzyme:solid ratio, 20 h, pH 5, 50°C], filtration [0.2-μm membrane pore size], atomization [130/85°C for inlet/outlet temperature] (Martín-Diana et al., 2021). As a consequence of these treatments, starch was partially reduced in SPD. The formation of considerable amounts of maltose (Table 1) and the reported secondary α-amylase activity of Ultraflo XL (Hu et al., 2014) suggest that starch reduction mostly occurred during the enzymatic treatment of WB.

When compared to WB, SPD showed lower TDF that was exclusively composed of SDF (Table 1) due to the solubilization of WB fiber components during the application of thermal and enzymatic treatments and further separation of the remaining IDF during the filtration process (Martín-Diana et al., 2021). The composition of SDF was noticeably modified because of enzymatic solubilization of WB non-starch polysaccharides that resulted in the accumulation of xylotriose and arabinoxylo- and feruloyl-oligosaccharides (Table 1; Supplementary Table 1). However, original amounts of β-glucans and raffinose family oligosaccharides remained unchanged. Ultraflo XL effects on DF of WB were also reflected by increase in di- and monosaccharides (maltose > glucose > fructose > sucrose > arabinose = cellobiose > xylose > galactose) (Table 1).

Protein content was significantly reduced in SPD (Table 1) probably because of formation of insoluble protein aggregates by thermal treatment and further removal by WB hydrolysate filtration (Harding et al., 2014). Similarly, the WB processing in this study decreased phytic acid content while increasing free P (Table 1). Autoclaving of the bran is known as an effective dephytinization treatment that could cause up to 96% reduction when performed at acidic pH for a long time of 1–2 h (Demir and Elgün, 2014; Onipe et al., 2021).

SPD showed higher TSPC and FA isomers (1.89- and 43.48-fold increase, respectively) than WB (Table 1) because of the combined action of thermal and enzymatic treatment with Ultraflo XL (Bautista-Exposito et al., 2020). Autoclaving solubilizes cell wall polysaccharides and favors the access of hydrolytic enzymes to cellulose and hemicellulose in the subsequent step of WB processing (Onipe et al., 2021). Atomization under the conditions used in this study was reported to have no influence on FA content (Martín-Diana et al., 2021). FA content in SPD reached 177 mg/100 g of dry powder (equivalent to the release 5 g of FA per kg of bran). This FA yield was within the range of reported values found in the literature (0.4–8.9. g/kg of bran) for similar approaches. For instance, Ferri et al. (2020) were able to achieve 1.5 g of FA /kg of WB by autoclave, steam explosion, and a combination of enzymatic treatments applied in two sequential steps. First, alcalase and Termamyl were employed to hydrolyze proteins and starch, respectively; then, Pentopan and feruloyl esterase were used for solubilization of DF and phenolic compounds of WB. More recently, Juhnevica-Radenkova et al. (2021) were able to obtain 8.6 g of FA/kg of WB with the single use of Vicozyme L for 24 h.

As expected, hydroxycinnamic acids were the most abundant phenolic group in which *trans*-FA was the main phenolic compound (accounting for 88.8% of TSPC), followed by lower amounts of *cis*-FA (Tables 3, 5). Consistent with our study (Si et al., 2020) and (Verma et al., 2009) identified FA as the major phenolic compound in WB hydrolysates. Hydroxybenzoic acids (4-hydroxybenzoic acid (i1), vanillic acid) flavonoids (apigenin diglucosides), and hydroxybenzaldehydes (vanillin) were also representative phenolic groups in SPD reported earlier (Martín-Diana et al., 2021).

Encapsulation of SPD with a commercial pea protein isolate (Pisane C_9_) at ratio 1:1 (w:w) explained most differences observed between the chemical composition of SPD and MEC. On the one hand, the amounts of β-glucan, TDF,SDF, total mono- and disaccharides, free P and TSPC (Table 1), and individual phenolic compounds (Table 5) in MEC were lower than the amounts observed in SPD because of the dilution effect caused by the use of the encapsulation agent in the atomization process (Table 1). On the other hand, the higher amounts of protein and phytic acid found for MEC (vs. SPD) were contributions of Pisane C_9_ (composed of 81.7% protein and 4.05% phytic acid) (Martín-Diana et al., 2021).

### Hydroxycinnamic Acids Are the Major Bioaccessible Phenolic Compounds of Wheat Bioprocessed Ingredients

Food matrix composition, food processing, and chemical structure influence the bioaccessibility of dietary phytochemicals (Shahidi and Pan, 2021). In line with this statement, the bioaccessibility of phenolic compounds varied across wheat derived ingredients (GERM, SPD, and MEC) and among different individual compounds.

Simulated digestion of GERM increased the total amounts of bioaccessible TSPC (41.69% relative increase vs. undigested sample) more extensively in the gastric phase (32% relative increase) than in the intestinal phase (9.5% relative increase) (Table 4). FA derivatives were the major bioaccessible phenolic compounds in GERM because of their high stability to digestion conditions as reported previously for barley and wheat sprouts (Aborus et al., 2018; Drawbridge et al., 2021). Maximum bioaccessible amounts of phenolic compounds were observed at the end of the intestinal phase for GERM, consistent with previous studies on *in vitro* digestion of conventional and bioprocessed (fermentation and enzymatic treatments) whole wheat bread (De Almeida et al., 2020). The main difference between our results and those obtained by previous studies regarding the *in vitro* digestion of sprouted cereal grains (oat, barley, and wheat) was higher release of phenolic compounds during the intestinal phase (Aborus et al., 2017, 2018). This difference may be attributed to the use of different digestion models. In fact, an obstacle in the interpretation of results from existing scientific literature is the large number of *in vitro* digestion models that differ on stages of digestion, conditions (enzymes, pH, electrolytes, and bile acids concentration, etc.), and modes of operation (static or dynamic) (Alminger et al., 2014). For this reason, in this article, we used a harmonized digestion protocol developed by the international INFOGEST network that sets parameters and conditions for static *in vitro* simulation of adult digestion suitable for food (Brodkorb et al., 2019).

There was a variation in the digestion behavior of individual phenolic compounds in GERM (Table 4). For instance, FA bioaccessibility increased gradually during all the stages of digestion in GERM probably because of physical, chemical, and enzymatic breakdown of the food matrix by the acidic environment of the stomach and pepsin action (Li et al., 2022). Moreover, in the intestinal phase, the esterase activity of lipases could contribute to the breakdown of covalent bonds and release of FA from the GERM food matrix (Shahidi and Pan, 2021). Gastric and intestinal digests contained too low amounts of 1-*O*-synapoyl-β-D-glucose to be detected possibly because of its transformation to other compounds either by hydrolysis or binding to other compounds such as proteins and starch. The bioaccessibility of apigenin and formonetin glycosides in GERM showed an opposite trend compared to that of FA. The conversion of flavonoid glucosides to aglycones induced by acidic conditions in the stomach might be a plausible mechanism that explains the lower content of these flavonoids at the end of gastric digestion (Ketnawa et al., 2021).

Unlike GERM, all phenolic compounds in SPD were in soluble forms as a result of the WB processing applied in this study. TSPC showed high stability during simulated digestion (<10% loss, Table 5) in SPD. *Trans*-ferulic acid was the major bioaccessible phenolic compound in SPD, with greater amounts during the gastric phase of digestion as compared with the end of intestinal digestion. A similar behavior was mostly observed for other hydroxycinnamic acids, hydroxybenzoic acids, apigenin glycosides, and vanillin during the digestion of SPD. Protein hydrolysis by pepsin and acidic pH during the gastric phase of digestion could have resulted in the disruption of hydrophobic interactions between free phenolic compounds and soluble proteins/peptides formed during WB processing. Previous studies have shown that caffeic and FAs are chemically unstable (oxidation or degradation) at the alkaline pH of pancreatic fluid (Chen et al., 2019; Ketnawa et al., 2021), explaining the decrease in phenolic compound bioaccessibility found for SPD after intestinal digestion.

As observed for MEC, 80% of TSPC were bioaccessible in the gastric phase, whereas greater bioaccessibility was observed at the end of the intestinal phase (120.14% of bioaccessibility, Table 5) that could be attributed to Pisane C_9_ contribution (0.1 g/100g of bioaccessible TSPC in the intestinal phase). Similar to SPD, FA isomers had good stability to digestion; therefore, hydroxycinnamic acids (with *trans*-FA as the most representative compound) were the major bioaccessible compounds in MEC. During digestion, the bioaccessibility of all the identified compounds showed a decreasing trend with the lowest values at the end of the intestinal phase. Interactions between phenolic compounds and food proteins is reported to be weakened during digestion (Chen et al., 2019). For example, the binding rate of oat aventhramides and caseins decreased from 70.8 to 38% after intestinal digestion, although new interactions may also appear between phenolic compounds and peptides (Chen et al., 2019). These peptides could be the carriers of phenolic compounds to small intestine where they could be metabolized by gut microbiota (Shahidi and Pan, 2021). All these effects may explain the lower bioaccessibility of individual phenolic compounds observed for MEC at either gastric or intestinal phase of digestion as compared to SPD. Consistent with our results, differences in food matrix composition have been associated to differential phenolic compound bioaccessibility in different cereal flours (whole meal wheat, rice, and corn). Seczyk et al. (2021) suggested that food matrices with high content of dietary fiber and proteins showed stronger interactions with polyphenols and lower digestibility (Seczyk et al., 2021). These findings also support the differences found between SPD and MEC, in which the higher protein content of the latter can be reflected by the higher affinity of phenolic acids with protein and their hydrolysis products (peptides).

Hydroxycinnamic acids, in particular ferulic acid or its derivatives, were the most abundant bioaccessible compounds during the digestion of the three bioprocessed wheat ingredients studied. Based on existing evidence, free FA is absorbed almost completely before reaching the colon (Zhao et al., 2003). In contrast, FA moieties in feruloyl arabinooligosaccharides or arabinoxylans could be released and then metabolized predominantly by colonic microbiota (Zhao et al., 2003). The main ferulic acid microbial colonic metabolites were reported to include 3-(3-hydroxyphenyl)propionic acid, dihydrocaffeic acid [3-(3,4-dihydroxyphenyl)propionic acid], 3-phenylpropionic acid, 3-(2-hydroxyphenyl)propionic acid (melilotic acid), and 3-(4-hydroxyphenyl)propionic acid (phloretic acid) (Anson et al., 2009; Koistinen et al., 2017). Interactions between cereal phenolic acids and gut bacteria may eventually exert functions linked to the observed health benefits of its consumption. For instance, ferulic acid metabolism has been reported to induce marked structural changes in the gut microbiota reducing the relative abundance of *Fimicutes, Erysipelotrichaceae*, and *Ileibacterium*, which were positively correlated with serum lipid levels in mice with atherosclerosis (Gu et al., 2021).

### Antioxidant Activity of Bioprocessed Wheat Ingredients Increased During Digestion

Dietary antioxidants may have a crucial function in the gastrointestinal tract by reducing the oxidative stress produced by respiratory bursts of immune cells activated by diet-related toxins and bacteria (Halliwell et al., 2005). Our results have demonstrated that undigested bioprocessed wheat ingredients studied herein were able to reduce free radicals through hydrogen (DPPH, ABTS, and ORAC) or electron donation (FRAP test) (Table 6). There was a variation in the overall antioxidant activity across bioprocessed ingredients (SPD > MEC > GERM) that was consistently associated to their TSPC, which is consistent with previous studies (Bautista-Exposito et al., 2020; Tome-Sanchez et al., 2020). As digestion progressed, a common increasing trend in most of the antioxidant parameters measured was observed for all the samples. The comparison among ingredients highlighted SPD (highest DPPH and FRAP values) as the most promising sample with the ability to prevent oxidative stress in the gastrointestinal tract.

In agreement with our results, Li et al. (2022) highlighted the increased radical scavenging activity of WB with different particle sizes during digestion (Li et al., 2022). Most studies have attributed this behavior to the higher bioaccessibility of total phenolic compounds during digestion (Sánchez-Velázquez et al., 2021; Li et al., 2022), as could be observed for GERM (in the gastric and intestinal phases, Table 4) and MEC (intestinal phase, Table 5). In contrast, this association could not be established for SPD in which bioaccessible TSPC were slightly reduced (<10%, Table 4). It has been demonstrated that in addition to the concentration of bioaccessible phenolic compounds, the pH of oral, gastric, and intestinal fluids play a role in the antioxidant activity of phenolics (Bouayed et al., 2011). The transition from acidic to alkaline environments enhances the antioxidant power of phenolics by causing deprotonation of hydroxyl moieties of aromatic rings, which could have contributed to the increased trend in the antioxidant activity observed during digestion of all the ingredients. Moreover, enzymatic hydrolysis in the stomach and intestine may contribute to the release of other compounds with antioxidant activity. For instance, the hydrolytic action of digestive enzymes in proteins during digestion releases peptides and free amino acids with radical scavenging activity (Vanvi and Tsopmo, 2016; Phongthai et al., 2018).

### Potential Anti-inflammatory Activity of Bioprocessed Wheat Ingredients Increased During *in vitro* Digestion

The bioprocessed wheat ingredients were able to inhibit the release of TNF-α and IL-6 by LPS-stimulated macrophages at the doses tested before and during simulated digestion (Table 7). This result indicated that the three ingredients studied have the potential to attenuate inflammation in the gut. Our study is in accordance with Gabriele et al. (2018), which demonstrated the anti-inflammatory effects of fermented whole wheat on TNFα-inflamed human intestinal epithelial cells (HT-29) (Gabriele et al., 2018). Particularly, the authors pointed out that free phenolic compounds released by fermentation reduced the expression of IL-8 and COX-2 inflammatory mediators through inhibition of the NF-κB signaling pathway.

The results indicated that there was a greater release of anti-inflammatory compounds during the intestinal phase of digestion than during the gastric phase (Table 7). Among the different factors involved in this effect, phenolic compounds might have played a role, since wheat hydroxycinnamic acids are able to inhibit the expression of IL-6 and TNF-α in LPS-activated RAW 264.7 macrophages (Luyen et al., 2014; Lampiasi and Montana, 2016). Nevertheless, other compounds might have contributed to the anti-inflammatory activity of bioprocessed ingredients. The presence of XOs in WB functional ingredients could promote their anti-inflammatory activity. In un-stimulated macrophages, xylooligosaccharides induced TNF-α, IL-1β, IL-6 and NO release, while inhibiting their production in a dose-dependent manner in LPS-stimulated RAW264.7 macrophages (Chen et al., 2012).

## Conclusions

This study demonstrate that WWG germination and WB bioprocessing result in promising outcomes for the creation of functional ingredients with improved nutritional profile. In particular, wheat germination was a useful method to enrich wheat flour in TDF, IDF, oligo- and monosaccharides, free phosphorous, and phenolic compounds, mainly FA derivatives, sinapoyl glucose, apigenin diglucosides, and formononetin. Similarly, the sequential combination of transformation (thermal and enzymatic treatments), separation (filtration), and drying (atomization) methods applied on WB served to obtain a soluble powder rich in SDF, oligo- and monosaccharides, and phenolic compounds that include FA as the most abundant compound followed by lower amounts of hydroxybenzoic acids, apigenin diglucosides, and vanillin. Another advantage of both bioprocessing methods was their effectiveness in dephytinization and increasing free P concentrations. Microencapsulation of SPD with Pisane C_9_ contributed to the increase in protein and phytic acid contents while at the same time diluting other SPD components.

Gastrointestinal digestion of GERM, SPD, and MEC revealed high stability of TSPC in both the gastric and intestinal phases. Hydroxycinnamic acids were the most bioaccessible compounds during digestion in the three bioprocessed wheat ingredients studied, although their bioaccessibility varied across ingredients. In this sense, the bioaccessibility of FA derivatives increased in GERM with progression of digestion while it was reduced in SPD and MEC up to the end of the intestinal phase. Microencapsulation of SPD with pea protein generally led to lower bioaccessible amounts of phenolic acids. Regarding biological properties, it was confirmed that digestion generally increased the ability of the ingredients to scavenge radicals and inhibit the production of proinflammatory cytokines. These findings highlight the potential of bioprocessing as a technological approach to valorize WWG and WB into added-value, and potential functional and nutraceutical properties of ingredients for innovative food applications.

## Data Availability Statement

The original contributions presented in the study are included in the article/Supplementary Material, further inquiries can be directed to the corresponding author/s.

## Author Contributions

CM-V: conceptualization and project administration. DR, EP, IJ-P, and IT-S: methodology. EP, IT-S, and IJ-P: software. CM-V, AM-D, DR, IT-S, EP, and IJ-P: validation. IT-S and IJ-P: formal analysis and data curation. IT-S, CM-V, EP, AM-D, IJ-P, DR, and JF: investigation. AM-D, CM-V, and DR: resources and supervision. AM-D, IT-S, and CM-V: writing-original draft preparation. AM-D, DR, IT-S, CM-V, EP, and JF: writing-review and editing. CM-V, AM-D, DR, IT-S, EP, IJ-P, and JF: visualization. JF, CM-V, and AM-D: funding acquisition. All authors have read and agreed to the published version of the manuscript.

## Funding

This research was funded by FEDER/Ministry of Science, Innovation and Universities-State Agency of Research (AEI/Spain and FEDER/UE) grant number AGL2017-83718-R. IT-S thanks to AEI/Spain and ESF/UE for her FPI fellowship (PRE2018-086464) and IJ-P to AEI/Spain and ESF/UE for his FPI fellowship (PRE2019-087824).

## Conflict of Interest

The authors declare that the research was conducted in the absence of any commercial or financial relationships that could be construed as a potential conflict of interest.

## Publisher's Note

All claims expressed in this article are solely those of the authors and do not necessarily represent those of their affiliated organizations, or those of the publisher, the editors and the reviewers. Any product that may be evaluated in this article, or claim that may be made by its manufacturer, is not guaranteed or endorsed by the publisher.
