# Supplementary material for: Bioprocessed Wheat Ingredients: Characterization, Bioaccessibility of Phenolic Compounds, and Bioactivity During in vitro Digestion
Source: Front Plant Sci. 2021 Dec 24;12:790898. doi: 10.3389/fpls.2021.790898 (PMC8740022; doi:10.3389/fpls.2021.790898)
Supplement: Supplementary file 2 [file Data_Sheet_2.docx]

Supplementary Material

# Supplementary Figures


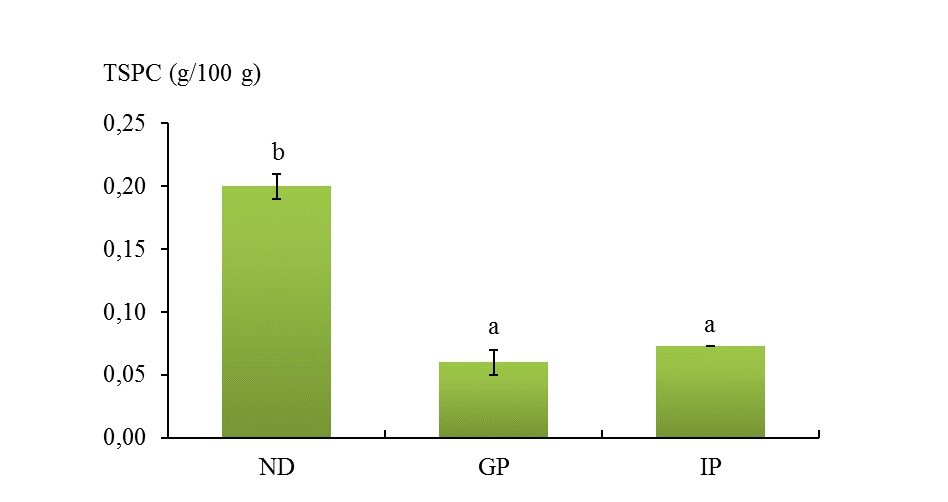


**Supplementary Figure 1.** Total soluble phenolic compounds (TSPC) in Pisane C_9_ during simulated digestion. Abbreviations: gastric phase (GP), intestinal phase (IP); non-digested (ND). Different lowercase letters denote significant differences between phases (one-way ANOVA, post hoc Dun- can’s test, *p* ≤ 0.05).
